# Information Provision Regarding Health-Related Direct-to-Consumer Genetic Testing for Dutch Consumers: An in-Depth Content Analysis of Sellers’ Websites

**DOI:** 10.3390/genes15040517

**Published:** 2024-04-20

**Authors:** Danny Bruins, Suzanne M. Onstwedder, Martina C. Cornel, Margreet G. E. M. Ausems, Marc H. W. van Mil, Tessel Rigter

**Affiliations:** 1 Section Community Genetics, Department of Human Genetics, Amsterdam Public Health Research Institute, Personalized Medicine, Amsterdam UMC, Vrije Universiteit Amsterdam, 1105 AZ Amsterdam, The Netherlands; d.bruins@amsterdamumc.nl (D.B.);; 2 Center for Health Protection, National Institute for Public Health and the Environment, 3721 MA Bilthoven, The Netherlands; 3 Department of Genetics, Division Laboratories, Pharmacy and Biomedical Genetics, University Medical Center Utrecht, 3584 CX Utrecht, The Netherlands; 4 Center of Education and Training, University Medical Center Utrecht, 3584 CX Utrecht, The Netherlands; 5 Center for Molecular Medicine, University Medical Center Utrecht, 3584 CX Utrecht, The Netherlands

**Keywords:** genetic testing, direct-to-consumer screening and testing, DTC-GT, internet, content analysis, SNP array, informed decision making

## Abstract

**Background**: Previous studies have suggested that information offered by sellers of health-related direct-to-consumer genetic tests (DTC-GTs) is often incomplete, unbalanced, or too difficult to understand. The extent to which this is the case for sellers accessible to Dutch consumers has not previously been studied. **Methods and Goals**: The present study aimed to assess the completeness, balance, readability, and findability of informational content on a selection of websites from several health-related DTC-GT sellers accessible to Dutch consumers. An in-depth content analysis was performed based on a recently published checklist outlining key items for policy guidance regarding DTC-GT services. **Results**: The information provided by sellers did not equally cover all aspects relevant to health-related DTC-GT service provision. The provided information was slightly unbalanced, with benefits of health-related DTC-GT usage being overemphasized compared to its risks and limitations. The readability of the provided information was low, on average requiring college education for proper understanding. A findability analysis showed that information concerning all themes is overall relatively evenly distributed across analyzed sellers’ websites. **Conclusions**: Information provision by assessed health-related DTC-GT sellers is suboptimal regarding completeness, balance, and readability. To better empower potential consumers to make an informed decision regarding health-related DTC-GT usage, we advocate industry-wide enhancement of information provision.

## 1. Introduction

Health-related direct-to-consumer genetic tests (DTC-GTs) are defined as commercially available DNA tests that inform consumers about their personal health and disease risks based on genetic make-up, without the involvement of a qualified health care professional (HCP) [1].

Recent studies have identified over 100 companies offering various types of health-related DTC-GTs, including nutrigenetic testing, pharmacogenetic testing, athleticism genetic tests, and overall disease risk genetic testing [2,3,4]. Specifically, a 2020 Dutch study focusing on the risks and benefits of health-related DTC-GTs for Dutch citizens revealed that the market for these tests is highly dynamic. It noted that sellers frequently enter and exit the market, or shift their focus to other types of DTC-GTs [5].

Since the emergence of health-related DTC-GTs, there has been an ongoing debate about whether their benefits outweigh their drawbacks. Often-named advantages include increased consumer autonomy and empowerment, enhanced societal knowledge about genetics and genomics, increased insight into disease risks, and the possibility of making positive lifestyle changes based on one’s genetic make-up [1,6,7]. Conversely, concerns have been raised about the tests’ unproven validity and utility, inadequate pre- and post-test counseling, and a lack of involvement of qualified HCPs. Combined, these issues could potentially lead to false expectations, misinterpretation of test results and consequent unnecessary medical and psychosocial impacts on consumers, and increased strain on the health care system if consumers decide to seek help from HCPs after unsettling DTC-GT results [6,8,9,10,11].

Given these concerns, and considering the lack of regulative and legislative consensus on DTC-GTs globally [4,12,13], it is crucial for interested individuals to make an informed decision about purchasing and using these tests. Balanced and high-quality information about genetic testing is essential for informed decision making [14]. Therefore, (potential) consumers should be provided with understandable information about the risks, benefits, and limitations of health-related DTC-GTs. It can be argued that sellers of these tests have both a moral responsibility and a legal obligation, as per European and national laws regarding genetic testing, to offer this information [5,15,16].

Several studies have suggested that information offered by DTC-GT sellers is often incomplete, unbalanced, or too difficult to understand [2,3,17,18]. These findings imply that consumers may not be able to make fully informed decisions based on the information provided by sellers, increasing the risk of unexpected or misunderstood test results. This study therefore aims to assess the completeness, balance, readability, and findability of information on several health-related DTC-GT sellers’ websites through in-depth content analysis. Additionally, how information is presented to consumers will be analyzed. Because this study is ultimately aiming to enable specifically Dutch citizens to make an informed decision regarding DTC-GTs, we selected websites that are most accessible to Dutch consumers for this in-depth analysis. By combining these data, the extent to which consumers encountering these sellers’ websites are enabled to make informed decisions regarding health-related DTC-GT usage will be gauged.

## 2. Materials and Methods

The process for evaluating the market and the criteria for the selection of websites for content analysis are described in detail in Supplementary Materials Document S1. The search terms utilized for the market analysis are shown in Supplementary Materials Document S2, Table S1. It should be emphasized that the purpose of this study was not to praise or criticize any sellers for their marketing strategies or the quality of information provided. To maintain objectivity, the results of the market analysis are anonymized, and the websites subjected to content analysis are encoded as company X, company Y, and company Z. The actual identities of these companies are known only to the authors.

### 2.1. Selection of Sellers for Content Analysis

The websites of three sellers offering health-related DTC-GTs were selected for content analysis. The selection was made based on the results of the market analysis (the likelihood of potential consumers to find the offer), geographical locations of the sellers’ headquarters and laboratories, genotyping technique, and the types of DTC-GTs offered. The chosen sellers were frequently encountered during the market analysis, were located in different countries, and offered health-related DTC-GTs based on SNP array technology.

### 2.2. Included Content from Sellers’ Webpages

For the content analysis, all consumer-focused webpages that were accessible within three or fewer clicks from the homepages of sellers’ websites and directly relevant to potential health-related DTC-GT consumers were collected as PDFs and uploaded onto MaxQDA (version 2022). Here, we distinguish a *website* as the complete online platform via which a DTC-GT is sold, and a *webpage* as one online page of a website, such as the homepage or product page. Webpages directed at informing HCPs, as well as those concerning other types of DTC-GTs such as tests to inform reproductive decision making (for example, carrier screening for autosomal recessive disorders), were excluded. This focus was chosen to omit the complexity of decision making in the context of reproduction and to purely focus on DTC-GTs for individual health decisions. Webpages only accessible after more than three clicks were also excluded on the basis that they were not easy for consumers to find. If pages other than the homepage contained more than five click-through pages that were accessible within three or fewer clicks from the homepage, such as FAQs or lists of drugs for pharmacogenetic assessment, five pages were randomly selected for content analysis. If pages contained a hundred or more click-through accessible pages within this criteria, 10% of these pages were randomly selected for content analysis.

### 2.3. Content Analysis Procedure Overview

**Figure 1:**
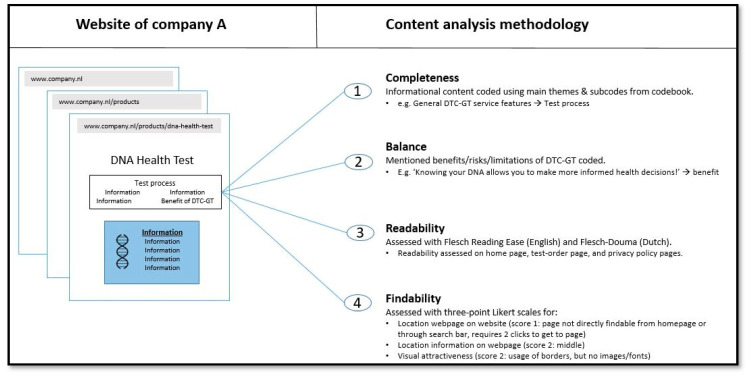
**Visual representation of content analysis workflow.** Pieces of information on each website were systematically coded to examine the completeness, balance, readability, and findability of information provided by selected sellers. For details on the codebook: see Supplementary Materials Document S2, Table S2.

The content analysis utilized a multi-layered approach for each website and webpage using MaxQDA (version 2022). Each website subjected to content analysis was coded independently by two coders (DB, SO, or TR), and discrepancies were discussed between the two coders for each website. The analysis aimed to assess the completeness, balance, readability, and findability of information on the sellers’ websites. The workflow for the content analysis is visually summarized in Figure 1. The methodologies used to assess the completeness, balance, readability, and findability of information are described in the following sections. 

#### 2.3.1. Assessing Completeness of Information

Firstly, the completeness of the information provided on sellers’ webpages was examined. This was achieved by coding the webpages’ content using a previously developed checklist for examining information provision by DTC-GT sellers developed by the Dutch National Institute for Public Health and the Environment as a basis [5]. This preliminary codebook was modified and subsequently validated by coders via (1) pilot coding of a website of a health-related DTC-GT seller excluded from the content analysis, and (2) pilot coding of webpages included in the content analysis. A total of 7 main themes were specified, with a total of 41 subcodes, as summarized in Table 1. Herein, a *main theme* is defined as a category of aspects that should be taken into account when evaluating the health-related DTC-GT offer [5]. These main themes are made up of individual *subcodes* that embody one of the aforementioned aspects. The final codebook including detailed code descriptions is available in Supplementary Materials Document S2, Table S2.

**Figure 2:**
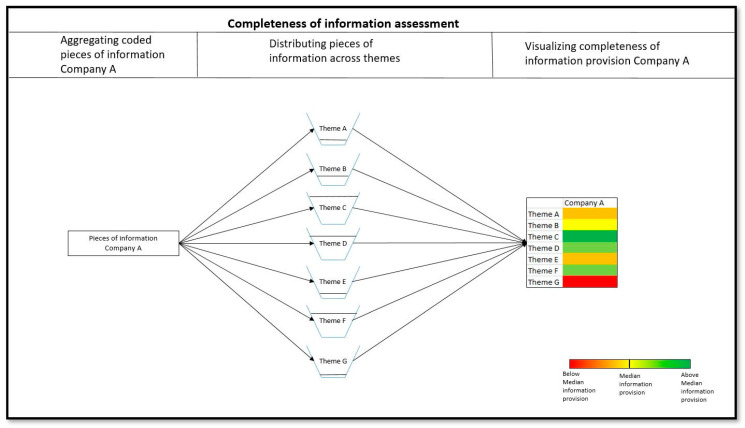
**Overview of workflow regarding assessment of completeness of information provision per seller.** Code usage per main theme across all coded pieces of information was assessed and visualized guided by median of observations.

To gauge the completeness of information provision per seller, code usage for each main theme was color-coded, guided by the median of the observations. The workflow of this assessment is schematically shown in Figure 2.

Due to our methodology which included coding not just text but also images, intercoder reliability (ICR) could not feasibly be calculated in MaxQDA (version 2022). For transparency, and to provide an indication of reliability regarding the completeness of information, individual subcode usage by each coder compared to their total individual code usage was evaluated for each website. Subsequently, the relative percentage usage of each main theme and subcode was compared between coders, and discrepancies of ≥5% between coders were flagged (see Supplementary Materials Document S2, Table S3). Moreover, to mitigate the impact of inconsistencies and coder-subjectivity, the number of codes used by both coders were averaged per main theme and subcode. These averages are the values reported in Section 3, with discrepancies highlighted to indicate reliability.

#### 2.3.2. Assessing the Balance of Information 

Secondly, the balance of provided information was assessed by analyzing the ratio of statements regarding the benefits of DTC-GTs to statements regarding risks and limitations of DTC-GTs. This approach provides an indication of informational balance, as previously demonstrated [18]. The codes for assessing the balance of information are shown in the fully modified codebook in Supplementary Materials Document S2, Table S2.

#### 2.3.3. Assessing the Readability of Information

Thirdly, readability was assessed using texts derived from selected pages of sellers’ websites, which were either in Dutch or English. English texts were assessed for readability using the Flesch Reading Ease (FRE) score [19], while the readability of Dutch texts was assessed using the Flesch–Douma readability score [20]. The latter method is based on the FRE score, but crudely adjusts for linguistic differences between English and Dutch, allowing for some degree of comparability between readability scores. Scores vary between 0 (not readable) and 100 (very easily readable), with standardized conversion tables described in the original publications introducing these formulas to determine the required reading grade level and description of style. For example, text excerpts with readability scores between 0 and 30 require the reading skills of a college graduate, whereas text excerpts with scores higher than 60 are deemed readable for everyone that completed primary school [19,20]. The texts used in the readability assessment were systematically derived from the same pages (homepage, test-ordering page, and privacy policy/terms and conditions pages) across websites to account for layout differences. 

#### 2.3.4. Assessing the Findability of Information

Finally, it is important to note that the completeness of readable and well-balanced information does not guarantee its effectiveness. It must also be easy for potential consumers to find the information. Therefore, we assessed the findability of information on the included webpages. Here, we define the *findability* of a piece of information as a combination of the accessibility and visual attractiveness of the information. Thus, to assess the findability of information, three three-point Likert scales were developed: one for the location of the webpage containing the piece of information on the website (*accessibility webpage*), one for the location of the piece of information on the respective webpages (*accessibility information*), and one to assess the visual attractiveness of the piece of information (*visual attractiveness*). These three three-point Likert scales and their exact definitions are also shown in the fully modified codebook in Supplementary Materials Document S2, Table S2. All pieces of information received scores ranging from 1 (lowest) to 3 (highest) on each scale. High scores in accessibility and visual attractiveness indicated better findability due to the direct influence of the location of information on its accessibility, and the faster processing of visual information compared to textual information [21,22].

An analysis of findability was performed using scores from these three Likert scales. For each Likert scale, a ‘poorly findable’ (pieces of information scoring a 1 on that respective Likert scale) and ‘easily findable’ (pieces of information scoring a 3) category were formed. Thus, a total of three poorly findable and three easily findable categories were formed. Subsequently, for each of these six respective categories, it was analyzed whether the distribution of information across the main themes differed from the overall distribution observed in the ‘completeness of information’ assessment. Given that each piece of information received a score on all three Likert scales, it is thus possible that a piece of information was deemed easily findable on one Likert scale and poorly findable on another.

**Figure 3:**
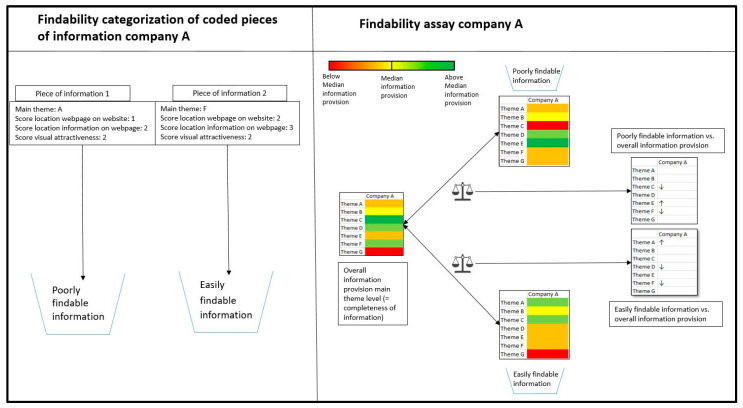
**Visual representation of findability assay methodology.** Coded pieces of information were categorized per seller as poorly findable or easily findable per Likert scale based on their assigned scores. As such, a total of six categories (three easily findable categories and three poorly findable categories) were defined. Subsequently, for each of the six categories, main theme usage was then compared between the easily findable and poorly findable categories and the overall information provision (resulting from the ‘completeness of information’ assessment). These comparisons revealed the distribution of themes associated with easily findable or poorly findable information on the sellers’ websites. Subsequently, code usage at the subcode level was utilized to interpret these differences. Note that the figure shows the ‘easily findable’ and ‘poorly findable’ categories for two different Likert scales. ↑: information corresponding to a theme more represented in a respective findability category (poorly or easily findable) compared to the overall information provision. ↓: information corresponding to a theme less represented in a respective findability category (poorly or easily findable) compared to the overall information provision.

The aim was to determine whether certain main themes were more prevalent in either the poorly findable or easily findable categories compared to their overall presence observed in the ‘completeness of information’ assessment. The main themes that were used more than the median level of main theme usage in the ‘completeness of information’ assessment, but less than the median level of main theme usage in either an easily findable or poorly findable category and vice-versa, are described in Section 3. Code usage at the subcode level for these main themes was utilized to explain these differences and interpret the implications of these differences in terms of information provision. The methodology for the findability assay is visualized in Figure 3.

## 3. Results

The anonymized companies X, Y, and Z whose websites were subjected to content analysis were highly findable during our market analysis focused on Dutch consumers (Supplementary Materials Document S1); they offer health-related DTC-GTs based on SNP array and are located in different countries. The results regarding the overall completeness, balance, and readability of information provided by these companies are summarized in Table 2 (main theme level) and Table 3 (subcode-level information provision), and subsequently described. The results regarding the findability of provided information per company are shown in Table 4 (information provision at main theme level for pieces of information with a maximum score (3) on at least one of the three Likert scales) and Table 6 (information provision at the main theme level for pieces of information with a minimum score (1) on at least one of the three Likert scales). Finally, Tables 5 and 7 compare the findability of information at the main theme level for easily findable (Table 5) and poorly findable (Table 7) information against the overall information provision shown in Table 2.

### 3.1. Completeness of Information

Table 2 and Table 3 show the similarities and variations in the completeness, balance, and readability of information provided by analyzed sellers. 

Table 2 reveals noticeable variation regarding the information provision across different main themes on sellers’ websites. Themes that were well represented across all sellers included ‘Information about Results, Interpretation, Consultation, and Endorsement’ and ‘General DTC-GT Service Features’. In contrast, remarkably less information was provided for themes like ‘Scientific Evidence’ and ‘Information about Potential Consequences of Performing DTC-GTs’, and especially ‘Informed Decision Making’. Additionally, the extent of information provision regarding main themes like ‘DNA Analysis and Quality Assurance’, ‘Scientific Evidence’, and ‘Information about Potential Consequences of Performing DTC-GTs’ varied substantially between companies.

Table 3 illustrates both the similarities and variations between sellers regarding information provision at the subcode level. Similar to the differences observed at the main theme level regarding the completeness of information provision, sellers’ websites also differed in the number of times pieces of information concerning specific subthemes were presented. 

This was especially striking for the theme ‘Information about Potential Consequences of Performing DTC-GTs’. Here, for all three companies, most of the available information is concentrated in just two out of seven subcodes, with the other five almost exclusively utilized at below-median levels. Considerable differences between companies are also noted for the main themes ‘Privacy and Data Management’ and ‘Information about Results, Interpretation, Consultation, and Endorsement’.

Regarding the theme ‘General DTC-GT Service Features’, it is interesting to note that for all sellers, information regarding the options to download your own raw data, or to opt-out of receiving certain results are considerably less well represented than, for example, information about what the buyer receives with buying the DTC-GT kit and the health aspects evaluated in the test.

Several subcodes such as ‘Information about result usability’, ‘Information about what the consumer receives with buying a DTC-GT kit’, and ‘Personal data management’ make up a substantial part of the total codes used for all sellers. In contrast, others like ‘Insurance impact’, ‘Analyzing the DNA of others without consent’, and ‘Active confirmation of informed decision’ are barely used, or not used at all. This indicates that the information provided by sellers does not equally cover all assessed aspects relevant to DTC-GTs.

### 3.2. Balance of Information

The analyzed sellers differed markedly regarding the balance of information. While one seller mentioned more risks and limitations than benefits of DTC-GT usage with a ratio of 5:2, the others mentioned more benefits than risks and limitations with a ratio of 3:1 and 5.5:1, respectively.

### 3.3. Readability of Information

The overall readability of analyzed sellers’ websites was comparable, ranging between 40 and 47. A closer examination of individual webpages revealed a trend: privacy pages (e.g., privacy policy, and terms and conditions) were notably less readable than test-ordering pages and homepages for all sellers.

### 3.4. Findability of Information

#### 3.4.1. Easily Findable Information vs. Overall Information Provision

An analysis of findability revealed that the distribution of easily findable information (scoring 3 on at least one of the three Likert scales) across the main themes (Table 4) was quite similar to the distribution of information across the main themes for the overall information provision (Table 2). 

However, some specific differences regarding the distribution of information across the main themes were observed among easily findable pieces of information compared to the overall information provision. These are shown in Table 5. Specifically, information related to the main theme ‘Information about Potential Consequences of Performing DTC-GTs’ was featured more prominently on both highly findable webpages, as well as in more visually attractive sections on the website of company Z. Analysis of the corresponding subcodes (Supplementary Materials Document S2, Table S4) revealed an increased representation of information related to the subcodes ‘Future health decision and behaviors’ and ‘Notion that results/impact may change due to future insights’ compared to their prevalence within the overall information provision.

Moreover, for company Y, the theme ‘Information about Potential Consequences of Performing DTC-GTs’ was more prevalent in visually attractive sections. This was mainly due to the increased presence of information related to the subcodes ‘Future health decision and behaviors’ and ‘Notion that results/impact may change due to future insights’. Additionally, the theme ‘Privacy and Data Management’ was also more prominently displayed in visually attractive sections on the website of company Y compared to its representation in the overall information provision. This difference was due primarily to a relative increase in information related to the subcodes ‘Genetic data management’, ‘Personal data management’, and ‘Privacy policy’.

Conversely, for company X, the findability analysis revealed that the main theme ‘DNA Analysis and Quality Assurance’ was less represented on both easily findable webpages and in visually attractive information sections. Although all associated subcodes were used less frequently in both of these situations compared to the overall information provision, the lack of information related to the subcode ‘Information about analyzed genes/variants’ was most noticeable.

#### 3.4.2. Poorly Findable Information vs. Overall Information Provision

The distribution of poorly findable pieces of information (scoring 1 on at least one of the three Likert scales) across the main themes (Table 6) showed few differences compared to the distribution of pieces of information across the main themes for the overall information provision (Table 2). 

However, some key differences were noted in the usage of the main themes in poorly findable information compared to their usage in the overall information provision. This comparison revealed fewer differences than the one between highly findable information and the overall information provision. These differences are also shown in Table 7. 

For company Y, a higher prevalence of information related to the main theme ‘Information about Potential Consequences of Performing DTC-GTs’ was observed on webpages that were harder to find, in comparison to its representation in the overall information provision. Analysis at the subcode level (Supplementary Materials Document S2, Table S5) revealed that this finding can be attributed to a relatively increased presence of information regarding the subcodes ‘Future health decisions and behaviors’ and ‘Notion that results/impact may change due to future insights’ on these webpages. Additionally, information regarding the main theme ‘Privacy and Data Management’ was more frequently encountered in sections of webpages that were harder to find. This correlated with more information related to the subcodes ‘Genetic data management’, ‘Personal data management’, and ‘Privacy policy’ being present in these sections as compared to in the overall information provision.

Conversely, for company Z, information regarding the main theme ‘Privacy and Data Management’ was less frequently encountered on webpages that were difficult to find. This trend could mainly be attributed to the presence of relatively few pieces of information pertaining to the subcodes ‘Genetic data management’, ‘Personal data management’, and ‘Data management certifications’. Likewise, information related to the main theme ‘General DTC-GT Service Features’ was not as prominent in hard-to-find sections on the webpages of company Z. The most noticeable aspect was the relative lack of information related to the subcode ‘Information about what the consumer receives with buying a DTC-GT kit’ in these sections.

## 4. Discussion

In this study, the websites of three representative sellers of health-related direct-to-consumer genetic tests (DTC-GTs) were analyzed using predetermined main themes and corresponding subcodes to assess the completeness, balance, readability, and findability of the information they provided. These three sellers were deemed representative based on the results of our market analysis focusing on potential Dutch consumers. The section of the discussion concerning the market analysis can be found in Supplementary Materials Document S1.

### 4.1. Completeness of Information

The content analysis revealed substantial variations in the amount of information provided across different themes, which was similar for all three companies. Information regarding the main themes ‘Scientific Evidence’, ‘Information about Potential Consequences of Performing DTC-GTs’, and especially ‘Informed Decision Making’ was noticeably less prevalent than information regarding the main themes ‘Information about Results, Interpretation, Consultation, and Endorsement’ and ‘General DTC-GT Service Features’. 

Additionally, a more in-depth inquiry utilizing subcodes revealed that information about what the consumer receives with buying the test, how the sellers manage a consumer’s personal data, and the usability of the results was far more prevalent than information concerning matters such as consent for testing, informed decision making, and the impact on health insurance for all analyzed sellers. Together, these findings indicate the presence of consistent variations in information provision from health-related DTC-GT sellers at both the main theme and subcode level.

There were also substantial variations between the sellers regarding the amount of information provided per main theme. This was especially noticeable for the themes ‘DNA Analysis and Quality Assurance’, ‘Scientific Evidence,’ and ‘Information about Potential Consequences of Performing DTC-GTs’. Likewise, at the subcode level, differences were observed in the themes ‘Privacy and Data Management’ and ‘Information about Results, Interpretation, Consultation, and Endorsement’. Subcodes such as ‘Possibility for consultation with genetic professional at company’, ‘Possibility for consultation with occupational professional at company’, and ‘Testing of minors’ received varying levels of attention among sellers.

Consistent with prior research [3,17], we found marked differences in the completeness of information concerning various aspects associated with health-related DTC-GT services. Notably, information beneficial for the companies in driving sales was featured more predominantly on the websites, such as the health features that are assessed in the test, the usability of test results, details about what the consumer will receive with buying the DTC-GT kit, and data management practices. In contrast, less information was provided on aspects that might cause potential consumers to reconsider purchasing the test, such as the risk for incidental findings, insurance complications, and non-medical impacts. This trend suggests that potential consumers are currently not being fully empowered to make an informed decision about using health-related DTC-GTs [14], as suggested previously [3,17,23].

### 4.2. Balance of Information 

There were remarkable differences among sellers regarding the balance of information. Company X mentioned 2 benefits for every 5 risks/limitations, whereas companies Y and Z mentioned approximately 5.5 and 3 benefits for each mentioned risk/limitation of DTC-GTs, respectively. These findings differ slightly from a previous study that found benefit statements outweighed risk/limitation statements by six to one across websites of 23 health-related DTC-GT sellers [18]. This suggests an improvement in the balance of information provided by health-related DTC-GT sellers over time, where especially company X stands out. However, further analysis showed that the majority of risk/limitation statements made by company X were in a visually unattractive, recurring disclaimer at the bottom of many analyzed webpages, considerably skewing the balance of information. This indicates that even if the balance of information provision may appear fair based on the ratio of benefit statements versus risk/limitation statements, potential consumers may still not be optimally empowered for informed decision making regarding health-related DTC-GT usage if the risk/limitation statements are hard to find.

### 4.3. Readability of Information

Readability analysis showed similar overall readability scores across sellers, ranging from 40 to 47, suggesting a ‘difficult’ level of readability, requiring ‘some college education’ to properly understand [19,20]. These findings are in line with previous studies assessing the literacy demands of various parts of health-related DTC-GT sellers’ websites [3,23]. Additionally, privacy-related pages were remarkably less readable than the homepages and test-ordering pages, potentially hindering informed decision making. Coders noted that pages with lower readability scores often harbored companies’ non-liability claims regarding the quality, reliability, and accuracy of their products and services, including any potential damages to consumers.

According to previous studies on overall literacy [24] and health literacy [25] in representative Dutch cohorts, a substantial number of potential Dutch consumers may experience difficulty in comprehending the information provided by health-related DTC-GT companies. This underscores the need to improve readability to optimally empower potential Dutch consumers for informed decision making regarding health-related DTC-GT usage.

### 4.4. Findability of Information

The in-depth findability analysis found that information on the main themes was generally evenly distributed between easily findable and poorly findable sections of sellers’ websites in terms of location and visual attractiveness. 

However, ten instances were identified where the main themes were differentially represented in these sections compared to the overall information provision. Among these, the theme ‘Information about Potential Consequences of Performing DTC-GTs’ was most often involved. In three out of the four times, we found variations in the presentation of this theme, as it was featured more predominantly in easily findable sections of information. While this may appear beneficial at first glance, the main subcodes within this theme involved future health consequences and insights, and the notion that results may change with future advancements, which were mainly presented in a positive light. These insights underscore that not all themes are equally findable, and that these imbalances could skew consumer perception and negatively impact informed decision making regarding health-related DTC-GT usage. However, the distribution of information across easily findable and poorly findable sections of sellers’ websites was generally quite even.

It should be noted that in light of informed decision making, it is unknown exactly how shortcomings in information provision impact informed decision making among Dutch citizens. Further studies investigating the needs and preferences regarding information provision concerning health-related DTC-GTs among Dutch citizens are warranted.

## 5. Limitations

When interpreting the results, several limitations of our study should be kept in mind. 

Because of the complexity and scale of the DTC-GT service market, we chose to limit our content analysis to the websites of three carefully selected health-related DTC-GT sellers that utilize SNP-based genotyping, vary in geographical locations of headquarters and laboratories, and cater to the Dutch public. This small sample size limits the generalizability of our results to the wider market. However, a strength is that the chosen sellers represent major health-related DTC-GT service providers that are among the ones most accessible to the Dutch public based on our market analysis. The provision of information on the websites of these companies may be representative of the most comprehensive information available to consumers, due to ample budget and greater liability exposure. Focusing on just three companies allowed for an in-depth analysis of the contents of the companies’ websites and the degree to which those contents empower potential consumers for informed decision making regarding health-related DTC-GT usage.

Not all pages of the selected sellers’ websites were analyzed, which means some information might have been overlooked. Excluded pages were either poorly findable (requiring more than three clicks from the homepage) or exhibited content so similar to other analyzed pages that we observed saturation. Thus, we deem it unlikely that this part of our methodology significantly impacted our results.

As for the actual content on selected sellers’ websites, only the presence of information was coded, whereas the actual quality and validity of information or the test itself were not assessed. Moreover, for all three sellers, we found and recoded several (nearly) identical pieces of information that recurred on all or most of the sellers’ webpages. These limitations may lead to an overestimation of the probability that consumers are receiving enough information to make truly informed decisions.

## 6. Summary, Impact, and Recommendations

An in-depth analysis of health-related DTC-GT vendors’ websites accessible to potential Dutch consumers uncovered disparities in the completeness, balance, and readability of the information provided by sellers. Information that could be seen as advantageous for sales was more abundant, whereas less emphasis was placed on areas that might deter consumers. Likewise, the potential benefits of health-related DTC-GTs were emphasized more often or were noticeably more findable compared to the risks and limitations. Additionally, the readability of the information was substandard, potentially hindering informed decision making by a substantial portion of the Dutch population. It is feasible that these findings can be generalized to the international DTC-GT market, but further research is warranted to substantiate this.

Overall, the findings of the present study highlight a need for significant improvements in the completeness, balance, and readability of the information provided by DTC-GT sellers to reduce the risk of unexpected medical, psychosocial, and societal impacts. To better empower potential consumers to make an informed decision regarding health-related DTC-GT usage, we advocate industry-wide enhancement of information provision.

## Figures and Tables

**Table 1:** **Main content themes and subcodes from codebook used in study.** Modified from [5].

Main Theme	Subcodes
General DTC-GT Service Features	Test process
	Test costs
	Assessed health features
	Information about what consumer receives with buying DTC-GT kit
	Option of raw data download
	Opt-out results
	Residency of company/lab
DNA Analysis and Quality Assurance	DNA test type
	Quality assurance
	Information about analyzed genes/variants
	Consumer background taken into account for analysis
Privacy and Data Management	Genetic data management
	Personal data management
	Privacy policy
	Sample management post-test
	Data/sample opt-out storing/selling/sharing
	Data management certifications
	Research participation
	Testing of minors
Scientific Evidence	Methods based on reliable scientific evidence
	Robustness of scientific evidence
	Scientific collaborations
Information about Results, Interpretation, Consultation, and Endorsement	Availability of demo report/hypothetical result report
	Information about assessed health features
	Information about result interpretation
	Information about result usability
	Possible actions upon getting results (consultation/interpretation)
	Possibility for consultation with genetic professional at company
	Possibility for consultation with occupational professional at company
	HCP consultation recommendation for test result discussion
	Referral for additional analysis
	Promotion of third-party products
Information about Potential Consequences of Performing DTC-GTs	Future health decisions and behaviors
	Insurance impact
	Impact on family members
	Impact on family relations
	Incidental/secondary/unexpected findings
	Consequences beyond medical purposes
	Notion that results/impact may change due to future insights
Informed Decision Making	Analyzing DNA of others without consent
	Active confirmation of informed decision

**Table 2:** **Overall information provision at the main theme level.** The results are shown per seller regarding the completeness (main theme level), balance, and readability of information provision. Values for the content codes of the main themes and the benefit versus risk/limitation ratio are averages from both individual coders’ scores. Values that are underlined, in italics, and in bold are values that showed a discrepancy of ≥5% between individual coders. Values are rounded up to the nearest whole number, and the benefit vs. risk/limitation balance ratio rounded to 2 decimals. Color coding for completeness (red–green): lowest value (1)—median value (138)—highest value (431). Color coding for balance (green–red): lowest value (0.44)—balance value (1)—maximum value (5.53). Color coding for readability (red–green): lowest possible value (0)—average value (50)—highest possible value (100).

	Company X	Company Y	Company Z
**General DTC-GT service features**	***253***	395	***371***
**DNA analysis & Quality Assurance**	***149***	70	200
**Privacy & Data Management**	***292***	133	205
**Scientific Evidence**	32	49	138
**Information about Results, Interpretation, Consultation, Endorsement**	257	234	431
**Information about potential consequences of performing DTC-GT**	66	136	78
**Informed Decision Making**	1	6	19
***Benefit vs. risk/limitation balance (ratio, value < 1 more risk/limitation, value > 1 more benefits)***	0.44	5.53	2.97
***Readability (0–100)***	45.5	46.3	40.7

**Table 3:** **Overall information provision at the subcode level.** The results per seller regarding the completeness and readability of information provision. The frequency of subcode usage per seller is presented. Values for the content subcodes are averages from both individual coders’ scores. Values that are underlined, in italics, and in bold are values that showed a discrepancy of ≥5% between individual coders. Values for the average subcode usage are rounded up to the nearest whole number. Combined values of subcode usage might not equal the main theme usage because of rounding averages and due to there being some main code usage when no subcode was deemed applicable. Color coding for subcode usage (red–green): lowest value (1)—median value (12)—highest value (197). Color coding for readability (red–green): lowest possible value (0)—average value (50)—highest possible value (100).

	Company X	Company Y	Company Z
**General DTC-GT service features**			
*Test process*	46	30	65
*Test costs*	18	84	12
*Assessed health features*	59	***130***	176
*Information about what consumer receives with buying DTC-GT kit*	100	***116***	102
*Option raw data download*	14	2	4
*Opt-out results*	13	0	6
*Residency of company/lab*	5	33	8
**DNA analysis & Quality Assurance**			
*DNA test type*	38	11	19
*Quality assurance*	47	25	87
*Information about analyzed genes/variants*	55	30	89
*Consumer background taken into account for analysis*	10	5	5
**Privacy & Data Management**			
*Genetic data management*	77	40	23
*Personal data management*	88	71	97
*Privacy policy*	***28***	13	7
*Sample management post-test*	11	3	2
*Data/sample opt-out storing/selling/sharing*	23	1	2
*Data management certifications*	3	1	52
*Research participation*	28	2	9
*Testing of minors*	35	4	13
**Scientific Evidence**			
*Methods based on reliable scientific evidence*	16	42	71
*Robustness of scientific evidence*	7	2	***63***
*Scientific collaborations*	9	5	5
**Information about Results, Interpretation, Consultation, Endorsement**			
*Availability of demo report/hypothetical result report*	2	1	31
*Information about assessed health features*	49	87	197
*Information about result interpretation*	35	5	46
*Information about result usability*	103	***104***	***82***
*Possible actions upon getting results (consult/interpretation)*	24	6	20
*Possibility for consultation genetic professional at company*	1	1	12
*Possiblity consultation occupational professional at company*	2	17	14
*HCP consult recommendation for test result discussion*	39	11	27
*Referral for additional analysis*	0	2	3
*Promotion of third party products*	4	3	1
**Information about potential consequences of performing DTC-GT**			
*Future health decisions & behaviors*	29	107	47
*Insurance impact*	3	2	2
*Impact on family members*	12	1	4
*Impact on family relations*	8	1	1
*Incidental/secondary/unexpected findings*	5	1	2
*Consequences beyond medical purposes*	8	2	3
*Notion that results/impact may change due to future insights.*	2	23	22
**Informed Decision Making**			
*Analyzing DNA of others without consent*	1	1	2
*Active confirmation of informed decision*	0	1	2
***Readability (0–100)***			
Homepage	50.8	48	42.6
Test order page	48.2	52	49.7
Privacy pages	37.5	39	29.7

**Table 4:** **The results per seller regarding the distribution of easily findable information across the main themes.** Information was considered easily findable when it had a high score (3) on at least one of the three Likert scales. For each Likert scale, an individual ‘easily findable’ category was formed. Likert scales evaluated different aspects of findability: the location of the specific webpage where information was located on the website (4.1), the placement of information on the webpage (4.2), and the visual attractiveness of the information (4.3). Values are averages from both individual coders’ scores. Values are rounded up to the nearest whole number. Color coding for the findability of the location of the webpage on the website (red–green): lowest value (0)—median value (19)—highest value (72). Color coding for the findability of the location of information on the webpage (red–green): lowest value (1)—median value (36)—highest value (241). Color coding for the findability of the visual attractiveness of information (red–green): lowest value (0)—median value (14)—highest value (106).

**4.1 Easily findable information per company: location of webpage on website**	**Company X**	**Company Y**	**Company Z**
**General DTC-GT service features**	41	61	58
**DNA analysis & Quality Assurance**	12	4	22
**Privacy & Data Management**	72	13	50
**Scientific Evidence**	7	6	12
**Information about Results, Interpretation, Consultation, Endorsement**	19	36	41
**Information about potential consequences of performing DTC-GT**	6	19	22
**Informed Decision Making**	0	4	0
**4.2 Easily findable information per company: location of information on webpage**	**Company X**	**Company Y**	**Company Z**
**General DTC-GT service features**	103	170	208
**DNA analysis & Quality Assurance**	36	28	76
**Privacy & Data Management**	99	30	49
**Scientific Evidence**	7	7	35
**Information about Results, Interpretation, Consultation, Endorsement**	71	90	241
**Information about potential consequences of performing DTC-GT**	20	40	33
**Informed Decision Making**	1	1	8
**4.3 Easily findable information per company: visual attractiveness of information on webpage**	**Company X**	**Company Y**	**Company Z**
**General DTC-GT service features**	35	89	82
**DNA analysis & Quality Assurance**	5	6	30
**Privacy & Data Management**	65	21	14
**Scientific Evidence**	6	8	6
**Information about Results, Interpretation, Consultation, Endorsement**	14	46	106
**Information about potential consequences of performing DTC-GT**	3	21	15
**Informed Decision Making**	0	1	1

**Table 5:** **An overview of median-based findability assay for easily findable pieces of information.** Pieces of information with the highest possible score (3) on at least one of the three findability Likert scales were considered easily findable. ↓: A main theme is presented relatively less often for a respective company within a respective highly findable Likert-scale category (webpage on website, information on webpage, and visual attractiveness of information) as compared to within the overall information provision. ↑: A main theme is presented relatively more often for a respective company within a respective highly findable Likert-scale category.

**5.1 Location webpage on website score 3 versus overall information provision**	**Company X**	**Company Y**	**Company Z**
**General DTC-GT service features**			
**DNA analysis & Quality Assurance**	↓		
**Privacy & Data Management**			
**Scientific Evidence**			
**Information about Results, Interpretation, Consultation, Endorsement**			
**Information about potential consequences of performing DTC-GT**			↑
**Informed Decision Making**			
**5.2 Location information on webpage score 3 versus overall information provision**	**Company X**	**Company Y**	**Company Z**
**General DTC-GT service features**			
**DNA analysis & Quality Assurance**			
**Privacy & Data Management**			
**Scientific Evidence**			
**Information about Results, Interpretation, Consultation, Endorsement**			
**Information about potential consequences of performing DTC-GT**			
**Informed Decision Making**			
**5.3 Visual attractiveness information on webpage score 3 versus overall information provision**	**Company X**	**Company Y**	**Company Z**
**General DTC-GT service features**			
**DNA analysis & Quality Assurance**	↓		
**Privacy & Data Management**		↑	
**Scientific Evidence**			
**Information about Results, Interpretation, Consultation, Endorsement**			
**Information about potential consequences of performing DTC-GT**		↑	↑
**Informed Decision Making**			

**Table 6:** **The results per seller regarding the distribution of poorly findable information across the main themes.** Information was considered poorly findable when it had a low score (1) on at least one of the three Likert scales. For each Likert scale, an individual ‘poorly findable’ category was formed. Likert scales evaluated different aspects of findability: the location of the specific webpage where information was located on the website (6.1), the placement of information on the webpage (6.2), and the visual attractiveness of the information (6.3). Values are averages from both individual coders’ scores. Values are rounded up to the nearest whole number. Color coding for the findability of the location of the webpage on the website (red–green): lowest value (0)—median value (65)—highest value (315). Color coding for the findability of the location of information on the webpage (red–green): lowest value (0)—median value (65)—highest value (168). Color coding for the findability of the visual attractiveness (red–green): lowest value (0)—median value (59)—highest value (141).

**6.1 Poorly findable information per company: location of webpage on website**	**Company X**	**Company Y**	**Company Z**
**General DTC-GT service features**	114	244	244
**DNA analysis & Quality Assurance**	65	47	126
**Privacy & Data Management**	140	45	39
**Scientific Evidence**	12	21	117
**Information about Results, Interpretation, Consultation, Endorsement**	133	138	315
**Information about potential consequences of performing DTC-GT**	39	83	30
**Informed Decision Making**	1	0	2
**6.2 Poorly findable information per company: location of information on webpage**	**Company X**	**Company Y**	**Company Z**
**General DTC-GT service features**	96	128	63
**DNA analysis & Quality Assurance**	87	23	76
**Privacy & Data Management**	117	83	119
**Scientific Evidence**	12	25	74
**Information about Results, Interpretation, Consultation, Endorsement**	134	65	168
**Information about potential consequences of performing DTC-GT**	23	47	34
**Informed Decision Making**	0	4	3
**6.3 Poorly findable information per company: visual attractiveness of information on webpage**	**Company X**	**Company Y**	**Company Z**
**General DTC-GT service features**	119	110	78
**DNA analysis & Quality Assurance**	85	21	59
**Privacy & Data Management**	133	68	91
**Scientific Evidence**	14	16	54
**Information about Results, Interpretation, Consultation, Endorsement**	138	59	141
**Information about potential consequences of performing DTC-GT**	37	38	32
**Informed Decision Making**	0	3	11

**Table 7:** **An overview of median-based findability assay for poorly findable pieces of information.** Pieces of information with the lowest possible score (1) on at least one of the three findability Likert scales were considered poorly findable. ↓: A main theme is presented relatively less often for a respective company within a respective poorly findable Likert-scale category (webpage on website, information on webpage, and visual attractiveness of information), as compared to within the overall information provision. ↑: A main theme is presented relatively more often for a respective company within a respective poorly findable Likert-scale category.

**7.1 Location webpage on website 1 versus overall information provision**	**Company X**	**Company Y**	**Company Z**
**General DTC-GT service features**			
**DNA analysis & Quality Assurance**			
**Privacy & Data Management**			↓
**Scientific Evidence**			
**Information about Results, Interpretation, Consultation, Endorsement**			
**Information about potential consequences of performing DTC-GT**		↑	
**Informed Decision Making**			
**7.2 Location information on webpage 1 versus overall information provision**	**Company X**	**Company Y**	**Company Z**
**General DTC-GT service features**			↓
**DNA analysis & Quality Assurance**			
**Privacy & Data Management**		↑	
**Scientific Evidence**			
**Information about Results, Interpretation, Consultation, Endorsement**			
**Information about potential consequences of performing DTC-GT**			
**Informed Decision Making**			
**7.3 Visual attractiveness information on webpage 1 versus overall information provision**	**Company X**	**Company Y**	**Company Z**
**General DTC-GT service features**			
**DNA analysis & Quality Assurance**			
**Privacy & Data Management**			
**Scientific Evidence**			
**Information about Results, Interpretation, Consultation, Endorsement**			
**Information about potential consequences of performing DTC-GT**			
**Informed Decision Making**			

## Data Availability

Data beyond those in the article and in the Supplementary Materials will not be shared to protect the anonymity of the companies.

## Supplementary Materials

The following supporting information can be downloaded at: https://www.mdpi.com/article/10.3390/genes15040517/s1. Word Document containing Supplementary Materials: Document S1. Market Analysis (with additional references [26,27,28,29,30,31]); Document S2, containing Supplementary Table S1. Search terms & Booleans; Supplementary Table S2. Codebook; Supplementary Table S3. ≥5% pt discrepancies; Supplementary Table S4. Subcode usage EF info; Supplementary Table S5. Subcode usage PF info.
