# Supplementary material for: Information Provision Regarding Health-Related Direct-to-Consumer Genetic Testing for Dutch Consumers: An in-Depth Content Analysis of Sellers’ Websites
Source: Genes (Basel). 2024 Apr 20;15(4):517. doi: 10.3390/genes15040517 (PMC11049909; doi:10.3390/genes15040517)
Supplement: Supplementary file 1 [file genes-15-00517-s001.zip › 20240418_ Supplementary Materials Document S2 Table S1-S5.docx]

Information Provision Regarding Health-Related
Direct-to-Consumer Genetic Testing for Dutch Consumers: An In-Depth Content Analysis of Sellers’ Websites

# Supplementary Materials Document S2: Tables S1-S5

## Content

| **Title** | **Description** |
| --- | --- |
| S1. Search terms & Booleans | Utilized search terms and Boolean operators for health-related DTC-GT market analysis for Dutch consumers. |
| S2. Codebook | Codebook utilized for in-depth content analysis of selected sellers. |
| S3. ≥5%pt discrepancies | Instances where individual coders differed more than ≥5%pt in relative subcode/main theme usage. |
| S4. Subcode usage EF info | Information provision (easily findable information) per seller on subcode level |
| S5. Subcode usage PF info | Information provision (poorly findable information) per seller on subcode level |

## Table S1. Search terms & Booleans

| **Language** | **Search terms** | **Booleans** |
| --- | --- | --- |
| Dutch |  |  |
|  | Genetische test | "genetische test" OR "genetische zelftest" OR "DNA-test" OR "DNA-zelftest" OR "DNA test" OR "DNA zelftest" OR "Test genetica" OR "DNA thuistest" |
|  | Genetische test kopen | genetische test OR "genetische zelftest" OR "DNA-test" OR "DNA-zelftest" OR "DNA test" OR "DNA zelftest" OR "Test genetica" OR "DNA thuistest" AND "kopen" |
|  | Genetische zelftest |  |
|  | Genetische zelftest kopen |  |
|  | DNA-test |  |
|  | DNA-test kopen |  |
|  | DNA-zelftest |  |
|  | DNA-zelftest kopen |  |
|  | DNA test |  |
|  | DNA test kopen |  |
|  | DNA zelftest kopen |  |
|  | Test genetica |  |
|  | Test genetica kopen |  |
|  | DNA thuistest |  |
| English |  |  |
|  | Genetic test | "genetic test" OR "genetic testing" OR "genetic self-test" OR "genetic self-testing" OR "genetic self-testing at home" OR "DNA test" |
|  | Buy genetic test | "DNA testing" OR "DNA self-test" OR "DNA self-testing" OR "DNA self-testing at home" OR "DNA test kit" OR "genetic test kit" |
|  | Genetic testing | "genetic test" OR "genetic testing" OR "genetic self-test" OR "genetic self-testing" OR "genetic self-testing at home" OR "DNA test" AND "buy" |
|  | Genetic self-test | "DNA testing" OR "DNA self-test" OR "DNA self-testing" OR "DNA self-testing at home" OR "DNA test kit" OR "genetic test kit" AND "buy" |
|  | Buy genetic self-test |  |
|  | Genetic self-testing |  |
|  | Genetic self-testing at home |  |
|  | DNA test |  |
|  | Buy DNA test |  |
|  | DNA testing |  |
|  | DNA self-test |  |
|  | Buy DNA self-test |  |
|  | DNA self-testing |  |
|  | DNA self-testing at home |  |
|  | DNA test kit |  |
|  | Buy DNA test kit |  |
|  | Genetic test kit |  |
|  | Buy genetic test kit |  |

## Table S2: Codebook

| **Code System** | | |  |
| --- | --- | --- | --- |
| *Theme/Code* | | | *Description* |
| **General DTC-GT service features** | | | Theme describing general service features of selected DTC-GT that company sells |
|  | General DTC-GT service features\Test process | | Information about test process (entire 'consumer journey': process ranging from creating accounts for kit registration to taking material to receving results: e.g. how to take sample, how to send the sample, how test is performed, how/when results will be received etc.) |
|  | General DTC-GT service features\Test costs | | Cost of the test |
|  | General DTC-GT service features\Assessed health features | | Detailed description of health features assessed by test, e.g. features associated with disease risk, athleti-genetics, pharmacogenetics, nutrigenetics, dermato-genetics. For example: risk for diabetes, VO2 max, drug metabolism enzymes, dietary needs, lactose intolerance, sunlight exposure, 'dietary/fitness/disease markers'. |
|  | General DTC-GT service features\Information about what consumer receives with buying DTC-GT kit | | General information about what a consumer receives with buying the DTC-GT kit in the broadest sense of the word. Such as the physical contents of the kit (e.g. swabs, return-shipping material, etc.), types of reports customers get as an end-product from the DTC-GT (e.g. lifestyle advice in the form of dietary/exercise advice, information about health risks), and how to use these in their lives (e.g. in the form of recipe advice, exercise advice, lifestyle advice to prevent disease, or diagnostic reports). Excludes things that can be bought for additional cost (e.g. counseling, additional reports). |
|  | General DTC-GT service features\Option to download raw DNA-data after testing | | There is an option to download your raw DNA-data after performing the test |
|  | General DTC-GT service features\Opt-out | | Option to opt-out from receiving certain results |
|  | General DTC-GT service features\Residency of company/lab | | Place where company and labs are located |
| **DNA analysis and quality assurance** | | | Theme for information regarding how the company analyzes customers' samples & assures quality of the test. |
|  | DNA analysis and quality assurance\DNA test type | | Information about the type of DNA-test that is offered, e.g. SNP array, WES, WGS |
|  | DNA analysis and quality assurance\Quality assurance | | Company makes claims about analytical quality assurance, (e.g. through mentioning certifications of labs performing analysis such as ISO-/CLIA-/CAP-certification), or practical quality assurance (e.g. 'receiving replacement kit if your kit arrives damaged'). |
|  | DNA analysis and quality assurance\Information about analyzed genes/variants | | Information about how many/which genes/variants are analyzed by the test |
|  | DNA analysis and quality assurance\Consumer background taken into account for analysis | | Relevant (medical) background information of consumer is taken into account (e.g. consumer has to provide family history, personal history of disease) |
| **Privacy & Data Management** | | | Theme for information regarding how company safeguards consumers' privacy, and how consumers' data is managed |
|  | Privacy & Data Management\Genetic Data Management | | Management of consumers' genetic data by company (e.g. storage duration of data, selling/sharing of data with third parties, usage of data in research) |
|  | Privacy & Data Management\Personal Data Management | | Management of consumers' provided personal data by company (e.g. e-mail address, personal data provided when making an account, personal data provided via e.g. questionnaire upon buying kit) |
|  | Privacy & Data Management\Privacy policy | | There is a privacy policy present on the website |
|  | Privacy & Data Management\Sample management post-test | | Management of consumers' sample after DNA-analysis by company (e.g. storage duration, what happens to sample if company goes bankrupt, etc.) |
|  | Privacy & Data Management\Data/sample opt-out | | Information about options to withhold DTC-GT company from storing, sharing, and selling of sample/data |
|  | Privacy & Data Management\Data management certifications | | Company mentions having data management certifications (e.g. ISO-certification), guaranteeing data safety. |
|  | Privacy & Data Management\Research participation | | Company provides information regarding usage of consumer's data (either genomic data or data consumer provides the company with themselves) for research. |
|  | Privacy & Data Management\Testing of minors | | Company provides information about testing of minors (<18), either directly (e.g. mentioning overall terms & conditions for testing of minors), or indirectly (e.g. stating 'testing is only for adults over 18'). |
| **Scientific Evidence** | | | Theme for information regarding scientific evidence for test that company provides. |
|  | Scientific Evidence\Methods based on reliable scientific evidence | | Company claims test methods are based on reliable scientific evidence. |
|  | Scientific Evidence\Robustness of scientific evidence | | Company makes claims about robustness of scientific evidence underlying test (e.g. novel gene-disease interactions vs. well-establisched gene-disease interactions) |
|  | Scientific Evidence\Scientific collaborations | | Company claims collaborations with scientific partners (e.g. academic research groups, prominent researches from universities) |
| **Information Results, Interpretation, Consultation, Endorsement** | | | Theme for information about post-test aspects of DTC-GT: results, interpretation of results, consultation of professionals at company, and third party endorsement. |
|  | Information Results, Interpretation, Consultation, Endorsement\Availability of demo report/hypothetical result report | | Company has a demo report freely available on website that potential customers can access to see how & what kind of information they will receive by doing test. |
|  | Information Results, Interpretation, Consultation, Endorsement\Information about assessed health features | | Company provides information about assessed health features (e.g. disease burden, disease/trait prevalence, molecular disease mechanism) |
|  | Information Results, Interpretation, Consultation, Endorsement\Information about result interpretation | | Company provides information about how to interpret results from test (e.g. explains what increased risk of 1.3x means, explains what 'high risk' entails) |
|  | Information Results, Interpretation, Consultation, Endorsement\Information about result usability | | Company provides information about robustness of results (e.g. usability for health decisions, behavior changes, diagnostic/medical vs informative/educational purposes, mention of the influence of environmental factors, influence of ethnicity etc.) |
|  | Information Results, Interpretation, Consultation, Endorsement\Possible actions upon getting result (consult/interpretation) | | Company lists/suggests possible actions for consumer to take regarding help with interpretation & consultation upon receiving test results (e.g. actions to undertake such as contacting HCPs, contact company for (paid) interpretation by employee/qualified individual) |
|  | Information Results, Interpretation, Consultation, Endorsement\Possibility for consultation genetic professional at company | | There is an option to consult a genetic professional at the company (either for free or through additional pay) |
|  | Information Results, Interpretation, Consultation, Endorsement\Possibility consultation occupational professional at company | | There is an option to consult a non-geneticist occupational professional (e.g. dietician, personal trainer) at the company (either for free or through additional pay). |
|  | Information Results, Interpretation, Consultation, Endorsement\HCP consult recommendation for test result discussion | | There is a recommendation to consult a HCP to discuss test results. |
|  | Information Results, Interpretation, Consultation, Endorsement\Referral for additional analysis | | There are referrals to other companies/websites for additional analysis on raw DNA-data (so-called third-party analyzers) |
|  | Information Results, Interpretation, Consultation, Endorsement\Promotion of third party products | | Company mentions/recommends/promotes third party products (e.g. nutritional supplements, fitness equipment) on their website. |
| **Information about potential consequences of performing DTC-GT** | | | Theme for information about potential consequences of performing DTC-GT. |
|  | Information about potential consequences of performing DTC-GT\Future health decisions & behaviors | | Notion that DTC-GT results may inform/impact future health decisions/behavior for consumers is made on the website. |
|  | Information about potential consequences of performing DTC-GT\Insurance impact | | Notion that DTC-GT results may impact insurance policies (health insurance, life insurance, etc.) |
|  | Information about potential consequences of performing DTC-GT\Impact on Family Members | | Notion that DTC-GT results may impact family members (potentially similar health risks as uncovered for test-taker) |
|  | Information about potential consequences of performing DTC-GT\Impact on Family Relations | | Notion that DTC-GT results may shed light on (unknown) family relations (e.g. test-taker carries pathogenic variant, mother's allele is present but father's allele differs not only at pathogenic variant site, but throughout gene allele → non-paternity) |
|  | Information about potential consequences of performing DTC-GT\Incidental/secondary/unexpected findings | | The possiblity for incidental/secondary/unexpected findings for consumers through DNA-testing is mentioned by the company. |
|  | Information about potential consequences of performing DTC-GT\Consequences beyond medical purposes | | Information about the fact that DTC-GT may have other consequences beyond medical purposes (e.g. uncertainty about results, psycho-social impact) |
|  | Information about potential consequences of performing DTC-GT\Notion that results/impact may change due to tech advances | | Company states that results/impact of DTC-GT may change based on future insights/innovations (e.g. new GWAS studies) |
| **Informed Decision Making** | | | Theme for information regarding informed decision making in DTC-GT. |
|  | Informed Decision Making\Analyzing DNA of others without consent | | Website provides information that analyzing DNA of others without consent is ethically irresponsible and/or punishable? |
|  | Informed Decision Making\Active confirmation of informed decision | | Consumer has to actively confirm informed decision (e.g. through clicking 'accept' instead of scrolling through a long text which automatically assumes confirmed informed decision after reading without active confirmation) |
| **Benefit/Risk/Limitation statements** | | | If you use a code on a certain piece of information on the webpage, also attach 1 of the 3 subcodes mentioned underneath to indicated whether the piece of information mentions a benefit, risk, or limitation of DTC-GT, or whether it does not (neutral). |
|  | Benefit/Risk/Limitation/Neutral statements\Benefit | | Statement of a benefit of DTC-GT testing (e.g. disease prevention, consumer education, personalized medicine, informed (health) decisions, altruism, research participation, ensuring privacy as opposed to genetic tests in public health system) |
|  | Benefit/Risk/Limitation/Neutral statements\Risk | | Statement regarding a risk of DTC-GT (e.g. worry/anxiety (both warranted and unwarranted), potential for genetic discrimination, impact on family members) |
|  | Benefit/Risk/Limitation/Neutral statements\Limitation | | Statement regarding limitations of DTC-GT (e.g. results not to be used for clinical goals, fact that diseases are multi-factorial, and gaps in current scientific understanding limiting interpretation of DNA) |
| **Findability Rankings** | | | How findable is the information you've attached a code to? Add both a 'Location' and 'Visual' score. Note that descriptions at 'Visual' and 'Location' sub-codes will likely never fully fit for a given snippet of information, and that information blocks may fit in 2 sub-codes, or fall between 2 sub-codes. In this case, follow gut feeling for initial scoring and label with a 'DEBATE'-code for discussion, along with memo why you're doubting the code. Code descriptions can be fine-tuned then. |
|  | Findability Rankings\Visual | | How visually attractive is the information presented? Assessment based on how the info is presented: usage of images/visual effects (e.g. usage of different color to highlight section of webpage, usage of videos) to convey information, and how the text is presented compared to other text on the webpage (e.g. usage of bold/italic/underlined text, larger/smaller font, different font type, colored font).  Wrote codes for 2 approaches: 1. a 1-3 Likert scale, where each individual code gets a Visual-score code, and 2. a single 'Visual best findable code', that can be used once per analyzed webpage to annotate the piece of information that, from a visual perspective, is most appealing.   Note that descriptions at 'Visual' Likert-scale sub-codes will likely never fully fit for a given snippet of information, and that information blocks may fit in 2 sub-codes, or fall between 2 sub-codes. In this case, follow gut feeling for initial scoring and label with 'DEBATE'-code for discussion, along with memo why you're doubting the code. Also label 'DEBATE' if you think a certain characteristic of information should be taken along for 'Visual' scoring that is not yet mentioned in the description. |
|  |  | *Findability Rankings\Visual\Visual score 1* | No usage of images/visual effects/videos to convey information, no effort to highlight text and make it distinct from other information through font and/or making text bold/italic/underlined, no usage of borders or other physical distinction methods on webpage to highlight information. E.g. information is presented in a block of homogeneous text in long privacy policy. |
|  |  | *Findability Rankings\Visual\Visual score 2* | No/limited usage of images/visual effects/videos to convey information, limited effort to highlight text and make it distinct from other information through font and/or making text bold/italic/underlined, no/limited usage of borders or other physical distinction methods on webpage to highlight information. |
|  |  | *Findability Rankings\Visual\Visual score 3* | Good usage of images/visual effects/videos to convey information, good effort to highlight text and make it distinct from other information through font and/or making text bold/italic/underlined, good usage of borders or other physical distinction methods on webpage to highlight information. |
|  | Findability Rankings\Location webpage on website | | On what location on the website is the information presented? Assessment based on: number of clicks required to reach webpage containing the information and whether webpage is directly findable through e.g. a navigation bar, or whether you need to click through pages to find the page.  Note that descriptions at 'Location webpage on website' Likert-scale sub-codes will likely never fully fit for a given snippet of information, and that information blocks may fit in 2 sub-codes, or fall between 2 sub-codes. In this case, follow gut feeling for initial scoring and label with 'DEBATE'-code for discussion, along with memo why you're doubting the code. Also label 'DEBATE' if you think a certain characteristic of information should be taken along for 'Location webpage on website' scoring that is not yet mentioned in the description.  NOTE: this score is for the location of the webpage on the website, not for the information on the webpage. |
|  |  | *Findability Rankings\Location webpage on website\Location webpage on website score 1* | Webpage is not directly findable through homepage or navigation bar, requires clicking through a secondary page to get to webpage. Maximum of 3 total clicks from homepage allowed, webpages findable after those 3 clicks are not taken into account for this analysis. |
|  |  | *Findability Rankings\Location webpage on website\Location webpage on website score 2* | Webpage is directly findable through a navigation bar. |
|  |  | *Findability Rankings\Location webpage on website\Location webpage on website score 3* | Webpage is the homepage itself, or directly findable via links on homepage. |
|  | Findability Rankings\Location information on webpage | | Where on the webpage is the respective information located (top/middle/bottom)? Usage of borders or other physical distinction methods (e.g. font type, bold/underlined/italic text) on webpage to highlight the information?  Note that descriptions at 'Location information on webpage' Likert-scale sub-codes will likely never fully fit for a given snippet of information, and that information blocks may fit in 2 sub-codes, or fall between 2 sub-codes. In this case, follow gut feeling for initial scoring and label with 'DEBATE'-code for discussion, along with memo why you're doubting the code. Also label 'DEBATE' if you think a certain characteristic of information should be taken along for 'Location information on webpage' scoring that is not yet mentioned in the description.  NOTE: this score is for information on a webpage, not for location of the webpage on the website. |
|  |  | *Findability Rankings\Location information on webpage\Location information on webpage score 1* | Information is located on bottom of the page, information is present in a drop-down section of the webpage that has to be actively opened.  Information present on webpages findable after >3 clicks is not taken into account for this analysis. |
|  |  | *Findability Rankings\Location information on webpage\Location information on webpage score 2* | Information is located on middle of the page. |
|  |  | *Findability Rankings\Location information on webpage\Location information on webpage score 3* | Information is located on top of the page |

## Table S3: ≥5%pt discrepancies

| Company | Main theme/subcode | Difference in relative coding frequency percentages between coders (abs, perc. points) |
| --- | --- | --- |
| Company X | **General DTC-GT service features** | 6,00401503 |
| Company X | **DNA analysis & Quality Assurance** | 5,00139728 |
| Company X | **Privacy & Data Management** | 8,96339133 |
| Company X | *Privacy policy* | 6,14089118 |
|  |  |  |
| Company Y | *Assessed health features* | 9,59773163 |
| Company Y | *Information about what consumer receives with buying DTC-GT kit* | 7,64575089 |
| Company Y | *Information about result usability* | 6,79735629 |
|  |  |  |
| Company Z | **General DTC-GT service features** | 5,63563824 |
| Company Z | *Robustness of scientific evidence* | 5,54776706 |
| Company Z | *Information about result usability* | 5,48317171 |

## Tables S4: Subcode usage EF info.

Color coding (red-green): lowest value-median value-highest value

| *: average values for both coders, values for subcode-usage rounded up to nearest whole number. |  |  |  |
| --- | --- | --- | --- |
| **Easily findable information (location of webpage on website score 3) per company on subcode level*** | **Company X** | **Company Y** | **Company Z** |
| **General DTC-GT service features** |  |  |  |
| *Test process* | 5 | 3 | 10 |
| *Test costs* | 10 | 13 | 7 |
| *Assessed health features* | 6 | 16 | 6 |
| *Information about what consumer receives with buying DTC-GT kit* | 18 | 24 | 32 |
| *Option raw data download* | 1 | 1 | 0 |
| *Opt-out results* | 3 | 0 | 2 |
| *Residency of company/lab* | 0 | 4 | 2 |
|  |  |  |  |
| **DNA analysis & Quality Assurance** |  |  |  |
| *DNA test type* | 6 | 0 | 3 |
| *Quality assurance* | 4 | 2 | 12 |
| *Information about analyzed genes/variants* | 2 | 1 | 5 |
| *Consumer background taken into account for analysis* | 1 | 1 | 2 |
|  |  |  |  |
| **Privacy & Data Management** |  |  |  |
| *Genetic data management* | 19 | 4 | 10 |
| *Personal data management* | 18 | 7 | 20 |
| *Privacy policy* | 10 | 2 | 1 |
| *Sample management post-test* | 3 | 0 | 0 |
| *Data/sample opt-out storing/selling/sharing* | 5 | 0 | 1 |
| *Data management certifications* | 2 | 0 | 10 |
| *Research participation* | 15 | 0 | 6 |
| *Testing of minors* | 2 | 1 | 4 |
|  |  |  |  |
| **Scientific Evidence** |  |  |  |
| *Methods based on reliable scientific evidence* | 4 | 5 | 7 |
| *Robustness of scientific evidence* | 1 | 0 | 5 |
| *Scientific collaborations* | 2 | 2 | 1 |
|  |  |  |  |
| **Information about Results, Interpretation, Consultation, Endorsement** |  |  |  |
| *Availability of demo report/hypothetical result report* | 1 | 0 | 3 |
| *Information about assessed health features* | 3 | 11 | 3 |
| *Information about result interpretation* | 1 | 2 | 4 |
| *Information about result usability* | 6 | 14 | 8 |
| *Possible actions upon getting results (consult/interpretation)* | 3 | 3 | 6 |
| *Possibility for consultation genetic professional at company* | 0 | 0 | 7 |
| *Possiblity consultation occupational professional at company* | 2 | 7 | 8 |
| *HCP consult recommendation for test result discussion* | 3 | 0 | 2 |
| *Referral for additional analysis* | 0 | 0 | 0 |
| *Promotion of third party products* | 0 | 1 | 0 |
|  |  |  |  |
| **Information about potential consequences of performing DTC-GT** |  |  |  |
| *Future health decisions & behaviors* | 5 | 16 | 12 |
| *Insurance impact* | 0 | 0 | 0 |
| *Impact on family members* | 0 | 0 | 2 |
| *Impact on family relations* | 0 | 0 | 0 |
| *Incidental/secondary/unexpected findings* | 0 | 0 | 0 |
| *Consequences beyond medical purposes* | 0 | 0 | 0 |
| *Notion that results/impact may change due to future insights.* | 1 | 4 | 8 |
|  |  |  |  |
| **Informed Decision Making** |  |  |  |
| *Analyzing DNA of others without consent* | 0 | 0 | 0 |
| *Active confirmation of informed decision* | 0 | 0 | 0 |

| Min: 0, Median:2, Max: 31 |  |
| --- | --- |

| *: average values for both coders, values for subcode-usage rounded up to nearest whole number. |  |  |  |
| --- | --- | --- | --- |
| **Easily findable information (location of information on webpage score 3) per company on subcode level*** | **Company X** | **Company Y** | **Company Z** |
| **General DTC-GT service features** |  |  |  |
| *Test process* | 25 | 11 | 40 |
| *Test costs* | 13 | 55 | 10 |
| *Assessed health features* | 19 | 57 | 89 |
| *Information about what consumer receives with buying DTC-GT kit* | 35 | 32 | 61 |
| *Option raw data download* | 9 | 2 | 4 |
| *Opt-out results* | 3 | 0 | 1 |
| *Residency of company/lab* | 1 | 4 | 5 |
|  |  |  |  |
| **DNA analysis & Quality Assurance** |  |  |  |
| *DNA test type* | 9 | 6 | 9 |
| *Quality assurance* | 15 | 6 | 16 |
| *Information about analyzed genes/variants* | 10 | 15 | 48 |
| *Consumer background taken into account for analysis* | 2 | 3 | 4 |
|  |  |  |  |
| **Privacy & Data Management** |  |  |  |
| *Genetic data management* | 27 | 12 | 7 |
| *Personal data management* | 31 | 12 | 23 |
| *Privacy policy* | 14 | 6 | 6 |
| *Sample management post-test* | 3 | 0 | 1 |
| *Data/sample opt-out storing/selling/sharing* | 6 | 0 | 1 |
| *Data management certifications* | 1 | 0 | 2 |
| *Research participation* | 16 | 0 | 4 |
| *Testing of minors* | 3 | 1 | 5 |
|  |  |  |  |
| **Scientific Evidence** |  |  |  |
| *Methods based on reliable scientific evidence* | 3 | 6 | 16 |
| *Robustness of scientific evidence* | 1 | 0 | 17 |
| *Scientific collaborations* | 3 | 1 | 2 |
|  |  |  |  |
| **Information about Results, Interpretation, Consultation, Endorsement** |  |  |  |
| *Availability of demo report/hypothetical result report* | 1 | 0 | 55 |
| *Information about assessed health features* | 21 | 42 | 108 |
| *Information about result interpretation* | 4 | 2 | 31 |
| *Information about result usability* | 23 | 36 | 28 |
| *Possible actions upon getting results (consult/interpretation)* | 8 | 1 | 5 |
| *Possibility for consultation genetic professional at company* | 1 | 1 | 4 |
| *Possiblity consultation occupational professional at company* | 0 | 4 | 5 |
| *HCP consult recommendation for test result discussion* | 14 | 4 | 3 |
| *Referral for additional analysis* | 0 | 1 | 3 |
| *Promotion of third party products* | 2 | 1 | 1 |
|  |  |  |  |
| **Information about potential consequences of performing DTC-GT** |  |  |  |
| *Future health decisions & behaviors* | 5 | 37 | 18 |
| *Insurance impact* | 2 | 0 | 1 |
| *Impact on family members* | 5 | 0 | 2 |
| *Impact on family relations* | 2 | 0 | 1 |
| *Incidental/secondary/unexpected findings* | 4 | 0 | 1 |
| *Consequences beyond medical purposes* | 3 | 0 | 1 |
| *Notion that results/impact may change due to future insights.* | 1 | 3 | 11 |
|  |  |  |  |
| **Informed Decision Making** |  |  |  |
| *Analyzing DNA of others without consent* | 1 | 0 | 2 |
| *Active confirmation of informed decision* | 0 | 0 | 1 |

| Min: 0, Median:4, Max: 108 |  |
| --- | --- |

| *: average values for both coders, values for subcode-usage rounded up to nearest whole number. |  |  |  |
| --- | --- | --- | --- |
| **Easily findable information (visual attractiveness of information score 3) per company on subcode level*** | **Company X** | **Company Y** | **Company Z** |
| **General DTC-GT service features** |  |  |  |
| *Test process* | 2 | 7 | 10 |
| *Test costs* | 5 | 18 | 6 |
| *Assessed health features* | 8 | 25 | 34 |
| *Information about what consumer receives with buying DTC-GT kit* | 15 | 35 | 31 |
| *Option raw data download* | 4 | 2 | 2 |
| *Opt-out results* | 2 | 0 | 0 |
| *Residency of company/lab* | 1 | 3 | 1 |
|  |  |  |  |
| **DNA analysis & Quality Assurance** |  |  |  |
| *DNA test type* | 1 | 0 | 3 |
| *Quality assurance* | 4 | 4 | 2 |
| *Information about analyzed genes/variants* | 0 | 2 | 25 |
| *Consumer background taken into account for analysis* | 0 | 1 | 1 |
|  |  |  |  |
| **Privacy & Data Management** |  |  |  |
| *Genetic data management* | 19 | 8 | 3 |
| *Personal data management* | 17 | 9 | 5 |
| *Privacy policy* | 6 | 4 | 1 |
| *Sample management post-test* | 4 | 0 | 0 |
| *Data/sample opt-out storing/selling/sharing* | 7 | 0 | 1 |
| *Data management certifications* | 1 | 0 | 1 |
| *Research participation* | 13 | 0 | 2 |
| *Testing of minors* | 0 | 1 | 3 |
|  |  |  |  |
| **Scientific Evidence** |  |  |  |
| *Methods based on reliable scientific evidence* | 3 | 6 | 4 |
| *Robustness of scientific evidence* | 0 | 1 | 2 |
| *Scientific collaborations* | 3 | 1 | 0 |
|  |  |  |  |
| **Information about Results, Interpretation, Consultation, Endorsement** |  |  |  |
| *Availability of demo report/hypothetical result report* | 1 | 0 | 29 |
| *Information about assessed health features* | 3 | 17 | 56 |
| *Information about result interpretation* | 0 | 2 | 11 |
| *Information about result usability* | 5 | 22 | 8 |
| *Possible actions upon getting results (consult/interpretation)* | 3 | 1 | 1 |
| *Possibility for consultation genetic professional at company* | 0 | 1 | 1 |
| *Possiblity consultation occupational professional at company* | 1 | 3 | 1 |
| *HCP consult recommendation for test result discussion* | 2 | 1 | 1 |
| *Referral for additional analysis* | 0 | 1 | 0 |
| *Promotion of third party products* | 0 | 1 | 0 |
|  |  |  |  |
| **Information about potential consequences of performing DTC-GT** |  |  |  |
| *Future health decisions & behaviors* | 3 | 18 | 12 |
| *Insurance impact* | 1 | 0 | 0 |
| *Impact on family members* | 0 | 0 | 1 |
| *Impact on family relations* | 0 | 0 | 0 |
| *Incidental/secondary/unexpected findings* | 0 | 0 | 0 |
| *Consequences beyond medical purposes* | 0 | 0 | 0 |
| *Notion that results/impact may change due to future insights.* | 0 | 4 | 2 |
|  |  |  |  |
| **Informed Decision Making** |  |  |  |
| *Analyzing DNA of others without consent* | 0 | 0 | 1 |
| *Active confirmation of informed decision* | 0 | 0 | 0 |

| Min: 0, Median:1, Max: 56 |  |
| --- | --- |

## Tables S5: Subcode usage PF info.

Color coding (red-green): lowest value-median value-highest value

| *: average values for both coders, values for subcode-usage rounded up to nearest whole number. |  |  |  |
| --- | --- | --- | --- |
| **Highly findable information (location of webpage on website score 1) per company on subcode level*** | **Company X** | **Company Y** | **Company Z** |
| **General DTC-GT service features** |  |  |  |
| *Test process* | 26 | 11 | 38 |
| *Test costs* | 1 | 53 | 3 |
| *Assessed health features* | 12 | 101 | 146 |
| *Information about what consumer receives with buying DTC-GT kit* | 51 | 59 | 53 |
| *Option raw data download* | 13 | 1 | 3 |
| *Opt-out results* | 9 | 0 | 3 |
| *Residency of company/lab* | 3 | 21 | 0 |
|  |  |  |  |
| **DNA analysis & Quality Assurance** |  |  |  |
| *DNA test type* | 18 | 8 | 11 |
| *Quality assurance* | 24 | 9 | 28 |
| *Information about analyzed genes/variants* | 18 | 28 | 84 |
| *Consumer background taken into account for analysis* | 6 | 2 | 3 |
|  |  |  |  |
| **Privacy & Data Management** |  |  |  |
| *Genetic data management* | 38 | 10 | 2 |
| *Personal data management* | 49 | 27 | 20 |
| *Privacy policy* | 9 | 7 | 1 |
| *Sample management post-test* | 6 | 0 | 0 |
| *Data/sample opt-out storing/selling/sharing* | 11 | 0 | 0 |
| *Data management certifications* | 1 | 0 | 12 |
| *Research participation* | 20 | 1 | 1 |
| *Testing of minors* | 8 | 0 | 2 |
|  |  |  |  |
| **Scientific Evidence** |  |  |  |
| *Methods based on reliable scientific evidence* | 5 | 18 | 61 |
| *Robustness of scientific evidence* | 5 | 0 | 55 |
| *Scientific collaborations* | 3 | 3 | 1 |
|  |  |  |  |
| **Information about Results, Interpretation, Consultation, Endorsement** |  |  |  |
| *Availability of demo report/hypothetical result report* | 1 | 0 | 29 |
| *Information about assessed health features* | 24 | 70 | 146 |
| *Information about result interpretation* | 11 | 1 | 41 |
| *Information about result usability* | 53 | 62 | 60 |
| *Possible actions upon getting results (consult/interpretation)* | 16 | 2 | 12 |
| *Possibility for consultation genetic professional at company* | 1 | 0 | 2 |
| *Possiblity consultation occupational professional at company* | 0 | 1 | 2 |
| *HCP consult recommendation for test result discussion* | 25 | 2 | 22 |
| *Referral for additional analysis* | 0 | 0 | 3 |
| *Promotion of third party products* | 4 | 1 | 1 |
|  |  |  |  |
| **Information about potential consequences of performing DTC-GT** |  |  |  |
| *Future health decisions & behaviors* | 16 | 71 | 23 |
| *Insurance impact* | 3 | 0 | 0 |
| *Impact on family members* | 9 | 0 | 1 |
| *Impact on family relations* | 4 | 0 | 1 |
| *Incidental/secondary/unexpected findings* | 2 | 0 | 0 |
| *Consequences beyond medical purposes* | 5 | 1 | 0 |
| *Notion that results/impact may change due to future insights.* | 1 | 12 | 6 |
|  |  |  |  |
| **Informed Decision Making** |  |  |  |
| *Analyzing DNA of others without consent* | 1 | 0 | 0 |
| *Active confirmation of informed decision* | 0 | 0 | 0 |

Min: 0, Median: 4, Max: 146

| *: average values for both coders, values for subcode-usage rounded up to nearest whole number. |  |  |  |
| --- | --- | --- | --- |
| **Highly findable information (location of information on webpage score 1) per company on subcode level*** | **Company X** | **Company Y** | **Company Z** |
| **General DTC-GT service features** |  |  |  |
| *Test process* | 19 | 11 | 10 |
| *Test costs* | 1 | 29 | 1 |
| *Assessed health features* | 27 | 26 | 36 |
| *Information about what consumer receives with buying DTC-GT kit* | 38 | 35 | 12 |
| *Option raw data download* | 5 | 1 | 1 |
| *Opt-out results* | 4 | 0 | 3 |
| *Residency of company/lab* | 3 | 28 | 1 |
|  |  |  |  |
| **DNA analysis & Quality Assurance** |  |  |  |
| *DNA test type* | 21 | 4 | 6 |
| *Quality assurance* | 25 | 12 | 57 |
| *Information about analyzed genes/variants* | 33 | 6 | 13 |
| *Consumer background taken into account for analysis* | 8 | 2 | 0 |
|  |  |  |  |
| **Privacy & Data Management** |  |  |  |
| *Genetic data management* | 28 | 22 | 5 |
| *Personal data management* | 37 | 45 | 56 |
| *Privacy policy* | 11 | 8 | 2 |
| *Sample management post-test* | 4 | 3 | 0 |
| *Data/sample opt-out storing/selling/sharing* | 10 | 1 | 0 |
| *Data management certifications* | 1 | 1 | 50 |
| *Research participation* | 16 | 2 | 4 |
| *Testing of minors* | 12 | 2 | 4 |
|  |  |  |  |
| **Scientific Evidence** |  |  |  |
| *Methods based on reliable scientific evidence* | 8 | 22 | 38 |
| *Robustness of scientific evidence* | 2 | 2 | 34 |
| *Scientific collaborations* | 3 | 2 | 2 |
|  |  |  |  |
| **Information about Results, Interpretation, Consultation, Endorsement** |  |  |  |
| *Availability of demo report/hypothetical result report* | 1 | 1 | 50 |
| *Information about assessed health features* | 32 | 17 | 36 |
| *Information about result interpretation* | 12 | 2 | 10 |
| *Information about result usability* | 60 | 31 | 36 |
| *Possible actions upon getting results (consult/interpretation)* | 10 | 3 | 23 |
| *Possibility for consultation genetic professional at company* | 0 | 0 | 1 |
| *Possiblity consultation occupational professional at company* | 2 | 9 | 1 |
| *HCP consult recommendation for test result discussion* | 16 | 2 | 12 |
| *Referral for additional analysis* | 0 | 0 | 0 |
| *Promotion of third party products* | 2 | 1 | 0 |
|  |  |  |  |
| **Information about potential consequences of performing DTC-GT** |  |  |  |
| *Future health decisions & behaviors* | 16 | 31 | 21 |
| *Insurance impact* | 1 | 2 | 1 |
| *Impact on family members* | 1 | 1 | 1 |
| *Impact on family relations* | 1 | 1 | 0 |
| *Incidental/secondary/unexpected findings* | 1 | 1 | 1 |
| *Consequences beyond medical purposes* | 3 | 2 | 2 |
| *Notion that results/impact may change due to future insights.* | 2 | 11 | 9 |
|  |  |  |  |
| **Informed Decision Making** |  |  |  |
| *Analyzing DNA of others without consent* | 0 | 1 | 1 |
| *Active confirmation of informed decision* | 0 | 1 | 0 |

Min: 0, Median: 3, Max: 60

| *: average values for both coders, values for subcode-usage rounded up to nearest whole number. |  |  |  |
| --- | --- | --- | --- |
| **Highly findable information (visual attractiveness of information score 1) per company on subcode level*** | **Company X** | **Company Y** | **Company Z** |
| **General DTC-GT service features** |  |  |  |
| *Test process* | 24 | 15 | 19 |
| *Test costs* | 1 | 21 | 1 |
| *Assessed health features* | 27 | 32 | 29 |
| *Information about what consumer receives with buying DTC-GT kit* | 54 | 17 | 23 |
| *Option raw data download* | 4 | 0 | 0 |
| *Opt-out results* | 3 | 0 | 4 |
| *Residency of company/lab* | 7 | 26 | 3 |
|  |  |  |  |
| **DNA analysis & Quality Assurance** |  |  |  |
| *DNA test type* | 24 | 3 | 4 |
| *Quality assurance* | 25 | 12 | 40 |
| *Information about analyzed genes/variants* | 28 | 4 | 16 |
| *Consumer background taken into account for analysis* | 9 | 2 | 1 |
|  |  |  |  |
| **Privacy & Data Management** |  |  |  |
| *Genetic data management* | 29 | 22 | 10 |
| *Personal data management* | 36 | 29 | 42 |
| *Privacy policy* | 19 | 10 | 2 |
| *Sample management post-test* | 4 | 3 | 2 |
| *Data/sample opt-out storing/selling/sharing* | 11 | 1 | 0 |
| *Data management certifications* | 1 | 1 | 25 |
| *Research participation* | 20 | 1 | 4 |
| *Testing of minors* | 14 | 3 | 7 |
|  |  |  |  |
| **Scientific Evidence** |  |  |  |
| *Methods based on reliable scientific evidence* | 5 | 12 | 21 |
| *Robustness of scientific evidence* | 5 | 1 | 32 |
| *Scientific collaborations* | 5 | 3 | 1 |
|  |  |  |  |
| **Information about Results, Interpretation, Consultation, Endorsement** |  |  |  |
| *Availability of demo report/hypothetical result report* | 1 | 1 | 39 |
| *Information about assessed health features* | 20 | 17 | 27 |
| *Information about result interpretation* | 21 | 3 | 5 |
| *Information about result usability* | 64 | 21 | 33 |
| *Possible actions upon getting results (consult/interpretation)* | 11 | 1 | 11 |
| *Possibility for consultation genetic professional at company* | 1 | 0 | 4 |
| *Possiblity consultation occupational professional at company* | 2 | 7 | 4 |
| *HCP consult recommendation for test result discussion* | 18 | 9 | 20 |
| *Referral for additional analysis* | 0 | 1 | 0 |
| *Promotion of third party products* | 1 | 0 | 0 |
|  |  |  |  |
| **Information about potential consequences of performing DTC-GT** |  |  |  |
| *Future health decisions & behaviors* | 18 | 26 | 18 |
| *Insurance impact* | 1 | 2 | 2 |
| *Impact on family members* | 4 | 1 | 1 |
| *Impact on family relations* | 5 | 1 | 0 |
| *Incidental/secondary/unexpected findings* | 3 | 1 | 2 |
| *Consequences beyond medical purposes* | 5 | 2 | 3 |
| *Notion that results/impact may change due to future insights.* | 1 | 7 | 7 |
|  |  |  |  |
| **Informed Decision Making** |  |  |  |
| *Analyzing DNA of others without consent* | 0 | 1 | 2 |
| *Active confirmation of informed decision* | 0 | 1 | 0 |

Min: 0, Median: 4, Max: 64
