# Supplementary material for: Information Provision Regarding Health-Related Direct-to-Consumer Genetic Testing for Dutch Consumers: An in-Depth Content Analysis of Sellers’ Websites
Source: Genes (Basel). 2024 Apr 20;15(4):517. doi: 10.3390/genes15040517 (PMC11049909; doi:10.3390/genes15040517)
Supplement: Supplementary file 1 [file genes-15-00517-s001.zip › 20240418_Supplementary Materials Document S1.docx]

Information Provision Regarding Health-Related
Direct-to-Consumer Genetic Testing for Dutch Consumers: An In-Depth Content Analysis of Sellers’ Websites

# Supplementary Materials Document S1: Market Analysis

Materials and Methods

Search terms

Search terms were either derived from previous DTC-GT market evaluation studies [1-4], or pragmatically conceived by the authors. Search terms were purposely designed on a non-professional level, and at a language proficiency level that could reasonably be used by consumers, since the aim was elucidation of the health-related DTC-GT market for Dutch citizens. Subsequently, individual search terms were combined into Dutch and English Boolean search terms using the ‘AND’ and ‘OR’ operators (**Supplementary Materials Document S2, Table S1.**) to refine searches and increase feasibility of the market analysis.

Search Engines

Searches were performed using some of the most-used search engines worldwide: Google Search, Microsoft Bing, and Yahoo Search. These search engines are the most-used ones worldwide across all platforms and for desktops specifically as of February 2023, with Google and Bing being the clear numbers 1 and 2. Yahoo Search was number 3, but closely followed by several other search engines such as Yandex [5]. For the Netherlands specifically, as of February 2023, the three most popular search engines across all platforms were Google Search, Microsoft Bing, and DuckDuckGo. However, for desktops in particular, the top three were Google Search, Microsoft Bing, and Yahoo Search [6]. Thus, Google Search, Microsoft Bing, and Yahoo Search were picked as search engines in an attempt to imitate a search by a potential Dutch consumer as well as possible, since this analysis would be performed on desktops in the Netherlands.

Search Strategy

Microsoft Edge was used as the Internet browser for the analysis. Searching was done using the Booleans shown in **Supplementary Materials Document S2, Table S1**. Before searching, the browser cache was fully cleared to prevent influence of algorithms on obtained search results as much as possible. Additionally, all searches were done in an InPrivate browser window with ‘Strict’ tracing prevention. Each Boolean was searched three times on each of the three search engines due to observed variability in search results for the same Boolean among different searches on the same search engine. Search engine settings were only manually changed from default if results appeared odd for Dutch citizens. For example, Microsoft Bing sometimes presumed the desktop that was performing the search to be located in Belgium instead of the Netherlands, thus yielding mostly Belgian websites as search results. Location settings were then manually chaged to ‘the Netherlands’.

Searches were carried out in February and March 2023. Search results, including ads, on the first two pages of each search for each search engine were considered for inclusion, since studies have shown that most search engine traffic stays on the first page, and only 1.1% goes beyond page 2 of search results [7]. Search results were documented per search engine, search number, and searched Boolean. They were also annotated for whether they were ads, or so-called organic hits that were results yielded by the actual search term.

Inclusion & Exclusion of search results

After documentation of search results for a given search, the contents of search results were subsequently studied by clicking on their respective links in the search engine. Search results were either included as validated hits (direct or indirect) for individual health-related DTC-GT sellers or wholesalers of these tests, or excluded from further analysis based on pre-determined criteria (**Table S1.1**). Excluded results were labeled with a reason for exclusion.

| **Inclusion criteria** | **Reason** |
| --- | --- |
| Direct hits to health-related DTC-GT sellers’ websites, regardless of where on website hit is (e.g. hit on blog post/learning hubs on seller’s website will be included as hit). | All these hits direct consumer directly to website of health-related DTC-GT seller. |
| Direct hits to websites stimulating consumers to buy health-related DTC-GT kits from certain sellers. Includes comparison websites (both sponsored and non-sponsored) for (health-related) DTC-GT sellers, and YouTube kit-comparison videos. | These websites aim to inform consumers about existence of (health-related) DTC-GT companies, and stimulate consumers (directly or indirectly) to buy kits from certain sellers. Sellers that are compared in these articles all receive 1 hit for these types of website, unless tests are compared on direct website of a DTC-GT seller (often biased towards favoring this seller), then only the direct seller received 1 hit. |
| Direct hits to websites of wholesalers of health-related DTC-GT kits. | Seller’s kits also reach consumer via wholesalers, thus wholesalers had to be included. If the number of kit-hits on wholesaler’s page was fifty or lower, the hit was counted for the specific sellers whose kit was sold. If the number of kit-hits on wholesaler’s page was higher than fifty, a singular wholesaler hit was counted. |
| Website hit has to be Dutch or English. | Many Dutch people speak Dutch and English, but other languages are less well-known. |
| Direct hits to lifestyle coaches’ websites, or other websites that re-sell, or have partnerships with sellers of health-related DTC-GTs. | It is feasible that consumers decide to buy such a test via lifestyle coaches [3], or that theycome into contact with these kits via other websites that re-sell, or have partnerships with sellers of health-related DTC-GTs. |
|  |  |
| **Exclusion criteria** | **Reason** |
| Direct hits to news articles that mention, or link to health-related DTC-GT sellers, but do not further stimulate buying through for example kit comparison. | No stimulation to ‘push’ consumer to buy health-related DTC-GT kits. |
| Direct hits to inaccessible website (error messages when entering website). | Consumer cannot order if website is inaccessible. |
| Hits to websites of sellers that only perform sequencing, without providing health-related reports based on sequencing results. | No full health-related DTC-GT service due to lack of providing health-related reports |
| Hits to website of third-party analyzers. | Websites do not sell health-related DTC-GT kits needed for genetic testing, only use sequencing data files that consumers already have. Only primary DTC-GT companies and secondary sellers (e.g. wholesalers) were considered for inclusion. |
| Hits to website of health-related DTC-GT seller whose test(s) cannot be shipped to the Netherlands. | Market analysis assessed health-related DTC-GT market for Dutch consumer. If the kit cannot be shipped to the Netherlands it is not interesting for potential Dutch consumer. Possibility for shipping to the Netherlands had to be deducible without making an account or giving personal information, otherwise seller was excluded. |
| Hits to website of health-related DTC-GT seller where test order has to be authorized by secondary person, such as a healthcare professional. | Excluded due to the need for authorization of DTC-GT by a secondary person, which contrasts with the definition of DTC-GT described in the Introduction of this article. |
| Hits to websites in language other than Dutch or English. | Many Dutch people speak Dutch and English, but other languages are less well-known. |
| Direct hits to DTC-GT sellers that do not sell health-related DTC-GTs. For example sellers of ancestry-only DTC-GT. | Non-health-related DTC-GTs were excluded from analysis. Consumers should be able to buy a health-related DTC-GT directly from the seller’s website without the need for e.g. purchasing an ancestry DTC-GT first, and only then being able to purchase a health-related analysis of their raw data (3^rd^ party analyzer service, performed by the same company). |

***Table S1.1: In- and exclusion criteria for search results.*** *Only included search results were used during data structuring.*

Data structuring

Validated hits for individual sellers of health-related DTC-GT kits, wholesalers, and websites providing indirect hits for direct sellers or wholesalers such as comparison articles, were documented per search engine, searched Boolean, and search number. Hits for sellers on websites of smaller wholesalers (fifty or less search results) and indirect-hit websites were counted as indirect hits for the sellers’ whose kits were sold/promoted on these websites. Hit types for individual sellers and wholesalers were divided into direct hits as ad, direct hits as organic search results, or indirect hits. Websites providing indirect hits for direct sellers or wholesalers were only eligible for inclusion if they were either a direct ad hit, or direct organic hit.

Next, all hit types for all individual sellers and larger wholesalers (more than fifty search results) were added together per Boolean to see which sellers were encountered most often per Boolean. Then, results from all Booleans in either Dutch or English were added up to see which sellers received hits most often in each language. Finally, results from all Booleans were added together to see which sellers and larger wholesalers were encountered most often during the whole analysis, and were thus deemed best-findable for potential Dutch health-related DTC-GT consumers. This workflow for the assessment of the health-related DTC-GT market for Dutch citizens is shown in **Figure S1.1.**


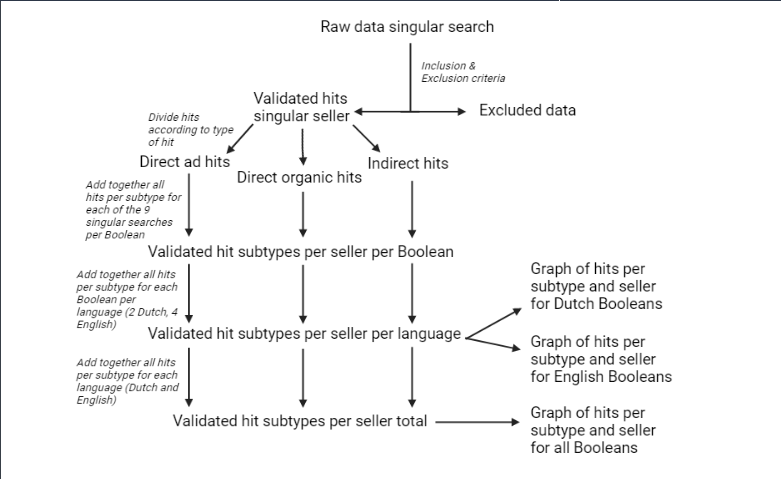

***Figure S1.1: Schematic workflow for the assessment of the health-related DTC-GT market for Dutch citizens.*** *Italic sections of text indicate performed actions. Singular seller could either be an individual health-related DTC-GT seller, or a large wholesaler.*

Results

The market analysis mainly served to inform the selection for the content analysis. For the Dutch Booleans, a total of 22 different sellers and wholesalers offering some form of health-related DTC-GT were identified. For the English Booleans, 24 different sellers were included. In total, 31 unique sellers and wholesalers of health-related DTC-GTs were identified during the searches (**Figure S1.2**). Most hits came in the form of indirect hits, e.g. through advertised websites comparing health-related DTC-GT kits of several sellers. Interestingly, the top-3 encountered sellers were the same for both Dutch and English Booleans (data not shown). The most-encountered seller after combining the results of the Dutch and English Booleans had 143 hits.


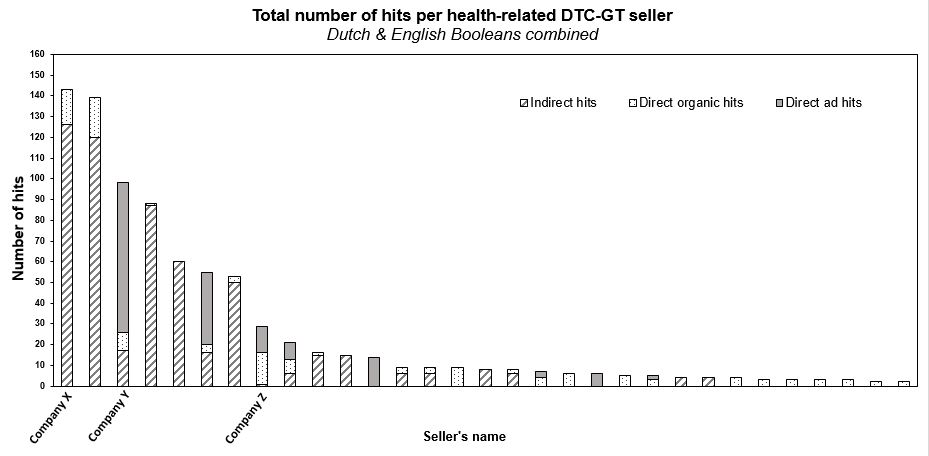


***Figure S1.2: Total number of hits per included health-related DTC-GT seller for Dutch and English Booleans combined.*** *Most hits were indirect hits, for example from websites comparing sellers of health-related DTC-GT kits.*

Discussion

Searches using Dutch Booleans yielded a total of 22 different sellers and wholesalers offering some form of health-related DTC-GT. For the searches using English Booleans, 24 different sellers were identified. In total, 31 unique sellers and wholesalers of health-related DTC-GTs meeting inclusion criteria were identified. Most hits came in the form of indirect hits, e.g. through advertised websites comparing health-related DTC-GT kits of several sellers. The most-encountered seller after combining the results of the Dutch and English Booleans had 143 hits.

Considerable differences were observed between the encountered sellers in this study and the sellers listed in a recent Dutch study that attempted to document the DTC-GT market for Dutch consumers [3]. This is likely in part due to a difference in aims. The present study only included sellers of health-related DTC-GTs that could be encountered using laymen level search terms, whereas the 2020 study included all known DTC-GT sellers without a focus on laymen’s search terms. However, the previously-identified rapid turnover of DTC-GT companies [3,8] could also play a significant role in the observed differences. Indeed, some of the sellers identified in 2020 are no longer active today. Additionally, several sellers whose webpages are still active, appear to be out of stock for their respective DTC-GT tests, hinting towards inactivity as well. These findings emphasize the need for regular re-assessment of the DTC-GT market.

One thing that stands out for the market analysis, is the proportion of indirect hits, where potential consumers are stimulated to access the websites of health-related DTC-GT sellers through another website (the ‘indirect hit’). These indirect hits often came from (sponsored) websites that compared several health-related DTC-GT sellers, and made recommendations for which ones to buy. Although direct hits could be considered as more convincing than indirect hits for the market analysis, comparison websites do provide a feasibly route through which consumers find health-related DTC-GT companies. Thus, they were scored similarly.

Another striking result is the fact that Dutch health-related DTC-GT sellers were also identified when searches using English Booleans were performed. However, these were only found as direct ad hits, and not also as direct organic hit or indirect hit like when searching with Dutch Booleans. This could be due to the web browser still registering that the searches were performed from the Netherlands, despite the taken precautions against the influence of algorithms on search results. As a result, ads for English search terms could be targeted towards Dutch people, leading to ads for Dutch health-related DTC-GT companies coming through when searching for English search terms.

Limitations

Market analysis

For the market analysis, we focused on sellers that sell a full health-related DTC-GT service (from analyzing a consumer’s sample to delivering a report with results) to potential consumers in the Netherlands. Thus, we excluded sellers of other types of DTC-GTs, and sellers that do not ship to the Netherlands. Additionally, the list of search terms for our market analysis was partly derived from previous publications, and partly pragmatically conceived by the authors. Thus, it is likely not to be exhaustive. As a result, we potentially failed to identify some health-related DTC-GT sellers that cater to the Dutch public.

As stated previously, we excluded DTC-GT sellers that did not offer a full health-related DTC-GT service from our market analysis. However, it is known that individuals from various populations utilize third-party interpretation tools (TPIs) to obtain a health report using raw data generated from non-health DTC-GTs that they performed earlier [9-11]. This provides a different avenue through which people can obtain health-related genetic information. Due to our market analysis methodology, we missed sellers of these non-health DTC-GTs. However, given that data regarding TPI usage in the Netherlands is lacking, the impact of this limitation is currently unknown. Monitoring the availability and usage of these TPIs in the Netherlands is an interesting avenue for further research.

1. Gollust, S.E.; Wilfond, B.S.; Hull, S.C. Direct-to-consumer sales of genetic services on the Internet. *Genet Med* **2003**, *5*, 332-337, doi:10.1097/01.GIM.0000076972.83711.48.

2. Hall, J.A.; Gertz, R.; Amato, J.; Pagliari, C. Transparency of genetic testing services for 'health, wellness and lifestyle': analysis of online prepurchase information for UK consumers. *Eur J Hum Genet* **2017**, *25*, 908-917, doi:10.1038/ejhg.2017.75.

3. Rigter, T.; Jansen, M.E.; van Klink-de Kruijff, I.E.; Onstwedder, S.M. *Kansen en risico's van DNA-zelftesten*; RIVM: RIVM, 2020.

4. Lachance, C.R.; Erby, L.A.; Ford, B.M.; Allen, V.C., Jr.; Kaphingst, K.A. Informational content, literacy demands, and usability of websites offering health-related genetic tests directly to consumers. *Genet Med* **2010**, *12*, 304-312, doi:10.1097/GIM.0b013e3181dbd8b2.

5. StatCounter. Search Engine Market Share Worldwide. Available online: <https://gs.statcounter.com/search-engine-market-share#monthly-202002-202303> (accessed on 1 February 2023).

6. StatCounter. Search Engine Market Share Netherlands. Available online: <https://gs.statcounter.com/search-engine-market-share/all/netherlands#monthly-202002-202303> (accessed on 1 February 2023).

7. DiAntonio, W. Do People Click on The Second Page of Google? Available online: <https://reputation911.com/do-people-click-past-the-first-page-of-google/> (accessed on 12 January 2023).

8. Phillips, A.M. 'Only a click away - DTC genetics for ancestry, health, love...and more: A view of the business and regulatory landscape'. *Appl Transl Genom* **2016**, *8*, 16-22, doi:10.1016/j.atg.2016.01.001.

9. Wang, C.; Cahill, T.J.; Parlato, A.; Wertz, B.; Zhong, Q.; Cunningham, T.N.; Cummings, J.J. Consumer use and response to online third-party raw DNA interpretation services. *Mol Genet Genomic Med* **2018**, *6*, 35-43, doi:10.1002/mgg3.340.

10. Nelson, S.C.; Bowen, D.J.; Fullerton, S.M. Third-Party Genetic Interpretation Tools: A Mixed-Methods Study of Consumer Motivation and Behavior. *Am J Hum Genet* **2019**, *105*, 122-131, doi:10.1016/j.ajhg.2019.05.014.

11. Metcalfe, S.A.; Hickerton, C.; Savard, J.; Terrill, B.; Turbitt, E.; Gaff, C.; Gray, K.; Middleton, A.; Wilson, B.; Newson, A.J. Australians' views on personal genomic testing: focus group findings from the Genioz study. *Eur J Hum Genet* **2018**, *26*, 1101-1112, doi:10.1038/s41431-018-0151-1.
